# Halogenated 3-Nitro-2*H*-Chromenes as Potential Agents Against Multidrug-Resistant Bacteria

**DOI:** 10.3390/antibiotics14030218

**Published:** 2025-02-21

**Authors:** Patrícia I. C. Godinho, Paula Pérez-Ramos, Yaiza Gabasa, Enmanuel Cornielle, Sara M. Soto, Raquel G. Soengas, Artur M. S. Silva

**Affiliations:** 1 LAQV-REQUIMTE, Department of Chemistry, University of Aveiro, 3810-193 Aveiro, Portugal; patricia.godinho@ua.pt; 2 Department of Organic and Inorganic Chemistry, University of Oviedo, and Instituto Universitario de Química Organometálica Enrique Moles, C/Julián Clavería 8, 33006 Oviedo, Spain; perezpaula@uniovi.es; 3 ISGlobal, 08036 Barcelona, Spain; yaiza.gabasa@isglobal.org (Y.G.); enmanuel.cornielle@isglobal.org (E.C.); sara.soto@isglobal.org (S.M.S.); 4 Facultat de Medicina i Ciències de la Salut, Universitat de Barcelona (UB), 08036 Barcelona, Spain; 5 CIBER Enfermedades Infecciosas (CIBERINFEC), Instituto de Salud Carlos III, 28028 Madrid, Spain

**Keywords:** chromenes, nitro derivatives, antibacterial, MDR bacteria, halogenated drugs

## Abstract

**Introduction/Objectives**: Nosocomial infections caused by *S. aureus* and *S. epidermidis* resistant strains are an important cause of morbidity and mortality worldwide. Due to the increasing rate of resistance to conventional antibiotics, the discovery of new antibiotic drugs is crucial to keep pace with the evolution of these pathogenic bacterial species. **Methods**: The 3-nitro-2*H*-chromene moiety is present in several compounds with potent antibacterial activity; based on these previous studies, we report herein the synthesis of 20 new 2-aryl-3-nitro-2*H*-chromene derivatives and the evaluation of their antibacterial potential in vitro. **Results**: Mono-halogenated nitrochromenes showed moderate anti-staphylococcal activity with MIC values of 8–32 μg/mL, whereas tri-halogenated 3-nitro-2*H*-chromenes displayed potent anti-staphylococcal activities with MIC values of 1–8 μg/mL. Notably, 2-(4-bromophenyl)-6-bromo-8-chloro-3-nitro-2*H*-chromene **5s** was the best antibacterial agent in the series against multidrug-resistant strains of *S. aureus* and *S. epidermidis* with MIC values of 4 μg/mL and 1–4 μg/mL, respectively. **Conclusions**: nitrochromene **5s** shows a good safety profile, so it can be considered as a lead for further development.

## 1. Introduction

The increasing number of infections caused by antibiotic-resistant bacterial pathogens over recent decades has become a critical global health problem [1]. For common bacterial infections, high rates of resistance against antibiotics frequently used to treat them have been observed worldwide, indicating that we are running out of effective antibiotics. If a sustained effort to contain antimicrobial resistance (AMR) is not undertaken, it is anticipated that by the year 2050, or before, there will be 10 million deaths caused by untreatable bacterial infections, well above the 1.8 million deaths due to cancer [2]. The clinical pipeline of new antimicrobials is dry. No new chemical classes of antibiotics have been introduced into the clinic in the past 20 years and large pharmaceutical companies have reduced or stopped their research and development due to the large investment and the high risk inherent in an antibiotic development project [3]. In 2021, the World Health Organization (WHO) identified only twenty-seven antibiotics in clinical development that address the priority pathogens, of which only two fulfil all the criteria to be considered as fully innovative: no cross-resistance, new chemical class, new target and new mechanism of action [4]. However, as most of the antibiotics under development are derivatives of the existing ones, the probability of generating bacterial resistance remains high. The urgent need of new drugs to fight against resistant bacteria has led researchers to mine the libraries of known pharmacophores in the search for new antimicrobial drugs [5].

Chromene derivatives are an essential class of oxygen-containing heterocyclic compounds that have attracted significant attention [6] due to their value as synthetic intermediates [7], their applications as pigments, cosmetics, and agrochemicals [8], and their wide range of biological activities [9,10,11,12,13] such as antidiabetic [14], antioxidant [15], anticancer [16], antiviral [17], and antibacterial activities [18,19]. Among chromenes, 3-nitro-2*H*-chromenes have been intensively investigated as potential antitumoral agents [20,21,22,23,24]. The antiproliferative effect of 3-nitrochromenes is related to their excellent inhibitory profile activity against thioredoxin reductase (TrxR) [25]. In this regard, TrxR has recently emerged as a viable and promising antibacterial drug target [26,27].

**Figure 1:**
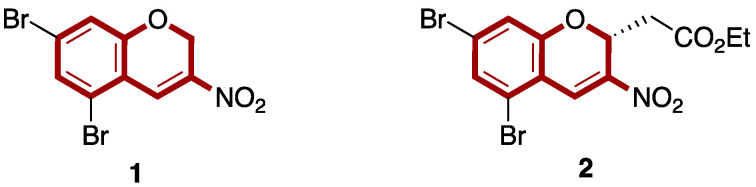
Antibacterial compounds containing the 3-nitro-2*H*-chromene scaffold.

However, there are limited reports in the literature dealing with the antibacterial activity of 3-nitro-2*H*-chromene derivatives [28,29]. Thus, Yan and co-workers reported the synthesis and characterization of a series of 3-nitro-2*H*-chromene derivatives. Their evaluation as antibacterial agents allowed the identification of derivative **1** as a potent antibacterial agent against *S. aureus* and methicillin-resistant *S. aureus* (MRSA) (MIC = 4 μg/mL) [28]. On the other hand, Li et al. described the synthesis and in vitro antibacterial evaluation of a library of twenty 2-alkyl 3-nitro-2*H*-chromenes. Among them, (3*R*)-ethyl-2-(6,8-dibromo-3-nitro-2*H*-chromene) acetate **2** proved to be the best antibacterial agent, with MBC values of 4 μg/mL against *S. aureus* and μg/mL against *S. epidermidis* (Figure 1) [29]. These preliminary results indicate that 3-nitro-2*H*-chromene represents a valuable pharmacophore for the development of new antibacterial drugs.

Taking into account the promising antibacterial profile of the 3-nitro-2*H*-chromene moiety, we report here the synthesis of a library of 2-aryl-3-nitro-2*H*-chromene derivatives and the evaluation of their antibacterial properties against a panel of resistant bacterial strains.

## 2. Results

### 2.1. Chemistry

An exhaustive literature search for methods described for the synthesis of 2-aryl-3-nitro-2*H*-chromenes **5** revealed that the simplest and most cost-effective method is the one-step *oxa*-Michael-Henry dehydration reaction developed by Nayak et al. [30]. However, the application of this strategy to the synthesis of a wide panel of nitrochromenes proved problematic, obtaining highly variable results depending on the electronic character of the substituents. Therefore, we introduced slight modifications to Nayak’s procedure, consisting of adding a small amount of acetonitrile to homogenize the mixture and increasing the reaction time. Thus, reaction between salicylaldehydes **3** and *trans*-nitrostyrenes **4** in the presence of DABCO as a catalyst and acetonitrile to homogenize the mixture afforded racemic 3-nitro-2*H*-chromenes **5a**–**t** (Table 1). The spectroscopic data of all nitrochromenes **5** supported their proposed structures (Supporting Information).

It was observed that the electronic character and position of the substituents in the aromatic rings of both salicylaldehydes **3** and nitroalkenes **4** have an influence on the reactivity. Thus, the yields are better the more pronounced the electron withdrawing character of the substituents in the aromatic ring of nitrostyrene is, due to increased electrophilicity of the β carbon of the nitroconjugated alkene. For that reason, halogenated nitrostyrenes **4d**–**f** give better yields than **4b**,**f**. On the other hand, the presence of electron withdrawing substituents in the salicylaldehydes decreases the nucleophilicity of the 2-hydroxy substituent, so the processes with mono-halogenated salicylaldehydes **3d**–**g** and di-halogenated salicylaldehydes **3m**–**o** are less efficient compared to the reaction carried out with unsubstituted salicylaldehyde **3a**. Reaction with nitrosalicylaldehyde **3h** did not take place, probably because the nitro group is a powerful electron withdrawing group. The reactions between the substituted di-*t*-butylsalicylaldehyde **3l** and nitroolefin **4a** did not occur, despite having two electron donor groups, possibly because of the steric hindrance caused by *t*-butyl groups located at positions 3 and 5 of the salicylaldehyde skeleton.

### 2.2. In Vitro Assays

The assayed chromenes showed bioactivity against Gram-positive bacteria but were non-effective against Gram-negative bacterial strains (Table 2). The bioassays revealed that the antibacterial activities of nitrochromene derivatives **5** are strongly dependent on the substitution pattern of the 3-nitro-2*H*-chromene core motif, ranging in activity from >128 μg/mL to 2 μg/mL. Thus, 3-nitro-2*H*-chromenes bearing electron donating groups in either ring A (**5b**, **5c**, **5h**–**j**) or ring C (**5k**, **5l**) resulted in almost no inhibition. However, mono-halogenated nitrochromenes (**5d**–**g**, **5m**–**o**) showed antibacterial activities higher than or similar to the unsubstituted nitrochromene derivative **5a**.

It was observed that, in particular, the enhanced antibacterial activity for the halogen substitution is slightly more pronounced in ring C. The incorporation of further halogen atoms proved beneficial for antibacterial activity. Thus, the presence of an additional halogen resulted in a significant improvement of the antibacterial activity of di-halogenated chromenes **5p** and **5q** against *S. epidermidis*. Moreover, tri-halogenated chromenes **5q**–**s** displayed higher antibacterial activity in the series. Notably, tri-halogenated 3-nitro-2*H*-chromene **5s** was shown to be the best potential antibacterial agent against *S. aureus* and *S. epidermidis* multidrug-resistant strains with MIC values of 4 μg/mL and 1–4 μg/mL, respectively.

We evaluated next the in vitro cytotoxicity against A549 lung epithelial cells of the most promising antibacterial agents, tri-halogenated 3-nitro-2*H*-chromene derivatives. The IC_50_ of compounds **5r**, **5s** and **5t** was 55.11 µM, 57.10 µM and 63.81 µM, respectively. Additionally, for a preliminary evaluation of ADME properties of the tri-halogenated 3-nitro-2*H*-chromene derivatives **5s**,**t,** an open access online in silico predictive model was used [31]. The model predicts a high gastrointestinal absorption and ability to cross the BBB for the three chromenes. Moreover, the predicted probability for the inhibition of P-glycoprotein and cytochrome CYP3A4 is low. Drug-likeness was also assessed and results showed that compounds **5s**,**t** comply with Lipinski and Veber rules, implying that they are probably orally bioavailable. 

## 3. Discussion

Infections caused by antibiotic-resistant *staphylococci* are a major threat for patients in health care centres. In this regard, *Staphylococcus aureus* and *Staphylococcus epidermidis* account for more than 20% of all health care-associated infections, affecting more than 250,000 patients around the globe each year [32]. Of special concern is the increasing incidence and prevalence of methicillin-resistant *S. aureus* (MRSA) bacteraemia, which exhibits high rates of morbidity and mortality and can lead to complications such as infective endocarditis or sepsis [33,34]. Antibiotic ceftaroline has been recently developed for the treatment of MRSA; although yet to be approved by the FDA, the results in the treatment of bacteraemia caused by MRSA are promising [35]. However, ceftaroline belongs to the well-known cephalosporins family, so the emergence of resistant strains is soon to be expected. Therefore, it is an urgent necessity of new molecules with new mechanisms of action. Thus, the development of novel antibacterial compounds for the management of minor and major infections due to antibiotic-resistant *staphylococci* is of paramount importance [36,37].

The nitro group (NO_2_) is an efficient scaffold when synthesizing new antibacterial molecules [38]. In fact, nitro-containing molecules are some of the first lines of treatment for common infections caused by several pathogenic bacteria [39,40]. The most accepted model for the antibacterial activity of nitro derivatives involves the in vivo reduction of the NO_2_ moiety, triggering the release of toxic intermediates such as nitroso and superoxide species [41]. Then, the reduced nitro species could bind covalently to DNA resulting in nuclear damage and cell death [42]. On the other hand, the nitro group itself also has a deep effect on the physicochemical and electronic properties of the drugs; it is a particularly electron-withdrawing moiety since the nitrogen has no lone pair, hence it bears a positive charge, favouring interactions with some amino acids of proteins [43]. In the present study, 20 nitrochromenes (5a–t) were efficiently synthesized by Michael–aldol reaction of salicylaldehydes 3 with nitrostyrenes 4. Biological assays showed that halogenated nitrochromenes (5d–g, 5m–o) were effective in vitro antimicrobial agents against Gram-positive *S. aureus* and *S. epidermidis*, indicating the importance of halogen atoms in determining the anti-staphylococcal potential. The presence of additional halogen atoms further potentiated the antibacterial activity. Thus, tri-halogenated compound **5s** was the most active, with MIC values of 4 μg/mL and 1–4 μg/mL against *S. aureus* and *S. epidermidis*, respectively. Moreover, tri-halogenated 3-nitro-2*H*-chromene derivatives 5r–p display low in vitro cytotoxicity, with IC_50_ values higher than 50 µM. In this regard, nitrochromene 5s presents a good safety profile, with a therapeutic index (TI) that exceeds the value of 10 for all the tested *S. aureus* and *S. epidermidis* strains.

Halogens are present in 25% of licensed drugs and 40% of actively tested lead compounds [44]. In fact, most of the pharmaceuticals referred to as “blockbuster drugs” are halogenated [45]. Halogen substituents can act as both electrophiles and nucleophiles, thus being capable of forming multiple covalent interactions with ligands [46,47]. Thus, the introduction of a carbon–halogen bond can influence the biological activities and can also have agonistic or antagonistic activities on bioactive targets due to their bulk [48]. Furthermore, adsorption, distribution, metabolism and excretion (ADME) parameters of compounds like drug binding affinity, membrane permeabilization and lipophilicity are influenced by halogen bonding [49]. Consequently, the roles and importance of halogens, especially fluorine and chlorine, in medicinal chemistry and FDA-approved drugs have been extensively studied [50,51,52,53,54]. Starting with the discovery of chloramphenicol in 1947 [55], halogens have now become prominent components of several classes of antibiotics and antibiotic scaffolds [56]. The addition of halogens to active compounds is now frequently used in the pharmaceutical industry as a strategy to potentiate the activity or selectivity of antimicrobial agents [57,58]. Furthermore, halogen incorporation was recently studied as an innovative means of restoring the activity of antibacterial agents against drug-resistant pathogens [59]. There are several previous studies where the presence of more halogens in the nitro derivative was a predictor of better biological activity. For example, the addition of two additional chloride atoms potentiates the activity against *S. aureus* of 4-nitro-substituted salicylic acid derivatives (MIC 4 μg/mL) compared to mono-chlorinated analogues (MIC 8 μg/mL) [60]. On the other hand, Li et al. showed that the (*R*)-ethyl 2-(3-nitro-2*H*-chromene) acetate bearing two bromine substituents is significantly more active against *Staphylococcus* sp. (*S. aureus*, MIC 4 μg/mL; *S. epidermidis*, MIC 8 μg/mL) than the corresponding mono-halogenated analogues (*S. aureus*, MIC 32–64 μg/mL; *S. epidermidis*, MIC 16–32 μg/mL) [29]. The results of the present study are in accordance with previous reports on the importance of halogenation in the antibacterial activity, confirming that the addition of halogens to nitro-derivatives could be a useful strategy to fight against antibacterial resistance.

Gram-negative pathogens selected in this study were predominantly non-susceptible to antibacterial action of the nitro-derivatives. In this regard, several researchers have reported that Gram-negative bacteria are considerably less susceptible to the antibacterial effects of nitro-compounds compared to Gram-positive pathogens [61,62,63]. This lower sensitivity was hypothesized to be associated with the outer membrane (OM) of the Gram-negative microorganisms, which creates an intrinsic barrier to the incorporation of the compound [64].

## 4. Materials and Methods

### 4.1. Chemistry

#### 4.1.1. General Procedures

All reagents employed during the development of this work are commercially available and were purchased from Chemosapiens S.L. NMR spectra were recorded in CDCl_3_ at 300 MHz for ^1^H and 75 MHz for ^13^C, with tetramethylsilane as internal standard for ^1^H and the residual solvent signals as standard for ^13^C. For the multiplicity of signals, the following abbreviations are used: s = singlet, bs = broad singlet, d = doublet, dd = double doublet, t = triplet, dt = double triplet, q = quatriplet, p = quintuplet and m = multiplet or unresolved, chemical shifts in ppm and coupling constant(s) in Hz. The values of the chemical shift of the signals in the NMR reports are in ppms. High-Resolution Mass Spectra (HRMS) were measured in ESI on a Bruker model Impact II.

#### 4.1.2. Synthesis of 3-Nitro-2*H*-Chromenes

A mixture of the appropriate salicylaldehyde **3a**–**g** (1 mmol), nitroalkenes **4a**–**f** (1 mmol) and DABCO (0.2 mmol) in acetonitrile (0.3 mL) was stirred while heating at 40 °C until reaction completion (≈3–6 h). After cooling down, the reaction mixture was diluted in ethyl acetate and washed with water and brine. The organic layer was extracted and dried over anhydrous Na_2_SO_4_. The crude was purified by flash chromatography on a silica gel column (70:1 hexane/ethyl acetate) and recrystallized in isopropanol to afford the pure 3-nitro-2*H*-chromenes **5a**–**t**. The characterization details of known chromenes **5a**–**f**, **5h**, **5j**–**r** match those reported in the literature [22,23,65,66,67,68,69].

3-Nitro-2-phenyl-2*H*-chromene (**5a**): Yellow solid. Yield: 60% (152 mg). ^1^H NMR (300 MHz, CD_3_COCD_3_): *δ* 8.32 (s, 1H), 7.61 (dd, *J* = 7.6, 1.7 Hz, 1H), 7.50–7.34 (m, 6H), 7.09 (td, *J* = 7.6, 1.1 Hz, 1H), 6.88 (d, *J* = 8.1 Hz, 1H), 6.65 (s, 1H). ^13^C NMR (75 MHz, CD_3_COCD_3_): *δ* 155.0, 147.1, 142.9, 141.8, 136.9, 129.7, 129.3, 128.8, 127.1, 120.4, 119.0, 117.8, 114.1, 73.8, 55.2.

6-Methoxy-3-nitro-2-phenyl-2*H*-chromene (**5b**): Yellow solid. Yield: 67% (190 mg). ^1^H NMR (300 MHz, CD_3_COCD_3_): *δ* 8.29 (s, 1H), 7.50–7.32 (m, 5H), 7.20 (d, *J* = 3.1 Hz, 1H), 6.98 (dd, *J* = 8.9, 3.1 Hz, 1H), 6.82 (d, *J* = 8.9 Hz, 1H), 6.59 (s, 1H), 3.80 (s, 3H). ^13^C NMR (75 MHz, CD_3_COCD_3_): *δ* 155.0, 147.1, 142.9, 141.8, 136.9, 129.7, 129.3, 128.8, 127.1, 120.4, 119.0, 117.8, 114.1, 73.8, 55.2.

6-Methyl-3-nitro-2-phenyl-2*H*-chromene (**5c**): Yellow solid. Yield: 63% (168 mg). ^1^H NMR (300 MHz, CD_3_COCD_3_): *δ* 8.25 (s, 1H), 7.48–7.33 (m, 6H), 7.26–7.16 (m, 1H), 6.78 (dd, *J* = 8.3, 0.7 Hz, 1H), 6.61 (s, 1H), 2.28 (s, 3H). ^13^C NMR (75 MHz, CD_3_COCD_3_): *δ* 151.2, 141.3, 137.1, 134.9, 132.0, 130.9, 129.7, 129.3, 128.8, 127.1, 118.3, 116.7, 73.9, 19.4.

6-Fluoro-3-nitro-2-phenyl-2*H*-chromene (**5d**): Yellow solid. Yield: 50% (136 mg). ^1^H NMR (300 MHz, CD_3_COCD_3_): *δ* 8.31 (s, 1H), 7.53–7.33 (m, 6H), 7.18 (ddd, *J* = 9.0, 8.4, 3.1 Hz, 1H), 6.91 (ddd, *J* = 9.1, 4.5, 0.9 Hz, 1H), 6.65 (s, 1H). ^13^C NMR (75 MHz, CD_3_COCD_3_): *δ* 158.1, 149.4, 142.3, 136.5, 129.5, 128.9, 128.7, 127.2, 120.6, 120.3, 119.5, 118.3, 116.4, 116.0, 74.1.

6-Chloro-3-nitro-2-phenyl-2*H*-chromene (**5e**): Yellow solid. Yield: 40% (115 mg). ^1^H NMR (300 MHz, CD_3_COCD_3_): *δ* 8.30 (s, 1H), 7.66 (d, *J* = 2.6 Hz, 1H), 7.48–7.41 (m, 2H), 7.41–7.33 (m, 4H), 6.89 (d, *J* = 8.7 Hz, 1H), 6.66 (s, 1H). ^13^C NMR (75 MHz, CD_3_COCD_3_): *δ* 151.9, 142.1, 136.6, 133.5, 129.8, 129.6, 128.9, 128.3, 127.2, 126.7, 120.0, 118.6, 74.3.

6-Bromo-3-nitro-2-phenyl-2*H*-chromene (**5f**): Yellow solid. Yield: 50%. (166 mg) ^1^H NMR (300 MHz, CD_3_COCD_3_): *δ* 8.29 (s, 1H), 7.79 (d, *J* = 2.5 Hz, 1H), 7.54–7.42 (m, 4H), 7.40–7.33 (m, 3H), 6.83 (d, *J* = 8.7 Hz, 1H), 6.66 (s, 1H). ^13^C NMR (75 MHz, CD_3_COCD_3_): *δ* 152.4, 142.1, 136.6, 136.4, 132.8, 129.6, 128.9, 128.2, 127.2, 120.5, 119.0, 113.8, 74.3.

3-Nitro-2-phenyl-6-trifluoromethoxy-2*H*-chromene (**5g**): Yellow solid. Yield: 59% (199 mg). Yellow solid. Yield: 59%. m.p. 73.3–73.8 °C. HRMS (ESI+) [M]^+^ calcd. for C_16_H_10_F_3_NO_4_, 337.0566; found, 337.0559. ^1^H NMR (300 MHz, CD_3_COCD_3_): *δ* 8.37 (s, 1H), 7.66 (dd, *J* = 3.0, 0.9 Hz, 1H), 7.52–7.45 (m, 2H), 7.44–7.33 (m, 4H), 7.00 (dd, *J* = 9.0, 0.9 Hz, 1H), 6.71 (s, 1H) ppm. ^13^C NMR (75 MHz, CD_3_COCD_3_): *δ* 151.9, 143.4, 142.3, 136.6, 129.7, 129.0, 128.4, 127.2, 126.7, 123.1, 122.2, 119.5, 118.8, 118.3, 74.4 ppm.

8-Methoxy-3-nitro-2-phenyl-2*H*-chromene (**5h**): Yellow solid. Yield: 65% (184 mg). ^1^H NMR (300 MHz, CD_3_COCD_3_): *δ* 8.28 (s, 1H), 7.50–7.43 (m, 2H), 7.40–7.34 (m, 3H), 7.18 (dd, *J* = 7.5, 1.6 Hz, 1H), 7.11 (dd, *J* = 8.2, 1.6 Hz, 1H), 7.02 (dd, *J* = 8.2, 7.5 Hz, 1H), 6.69 (s, 1H), 3.77 (s, 3H). ^13^C NMR (75 MHz, CD_3_COCD_3_): *δ* 148.8, 142.6, 141.3, 137.1, 129.6, 129.3, 128.8, 127.0, 122.4, 122.3, 119.1, 117.2, 73.7, 55.6.

8-Methyl-3-nitro-2-phenyl-2*H*-chromene (**5i**): Yellow solid. Yield: 52% (139 mg). m.p. 116.5–117 °C. HRMS (ESI+) [M]^+^ calcd. for C_16_H_10_NO_3_, 267.0896; found, 267.0889. ^1^H NMR (300 MHz, CD_3_COCD_3_): *δ* 8.30 (s, 1H), 7.51–7.33 (m, 6H), 7.26 (ddt, *J* = 7.6, 1.7, 0.8 Hz, 1H), 6.97 (t, *J* = 7.6 Hz, 1H), 6.70 (s, 1H), 2.12 (s, 3H) ppm. ^13^C NMR (75 MHz, CD_3_COCD_3_): *δ* 151.3, 141.0, 137.2, 135.4, 130.0, 129.3, 128.8, 128.6, 126.9, 126.2, 122.2, 118.2, 73.8, 14.7 ppm.

8-Ethoxy-3-nitro-2-phenyl-2*H*-chromene (**5j**): Yellow solid. Yield: 66% (196 mg). ^1^H NMR (300 MHz, CD_3_COCD_3_): *δ* 8.28 (s, 1H), 7.51–7.43 (m, 2H), 7.42–7.30 (m, 3H), 7.18 (dd, *J* = 7.6, 1.6 Hz, 1H), 7.10 (dd, *J* = 8.2, 1.6 Hz, 1H), 7.00 (dd, *J* = 8.2, 7.6 Hz, 1H), 6.69 (s, 1H), 4.01 (qd, *J* = 7.0, 3.3 Hz, 2H), 1.30 (t, *J* = 7.0 Hz, 3H). ^13^C NMR (75 MHz, CD_3_COCD_3_): *δ* 148.0, 143.0, 141.4, 137.1, 129.7, 129.3, 128.7, 127.0, 122.5, 122.5, 119.3, 118.9, 73.6, 64.7, 14.2.

2-(4-Methoxyphenyl)-3-nitro-2*H*-chromene (**5k**): Yellow solid. Yield: 71% (201 mg). ^1^H NMR (300 MHz, CD_3_COCD_3_): *δ* 8.29 (s, 1H), 7.60 (dd, *J* = 7.6, 1.7 Hz, 1H), 7.43–7.34 (m, 3H), 7.08 (td, *J* = 7.6, 1.1 Hz, 1H), 6.94–6.83 (m, 3H), 6.59 (s, 1H), 3.77 (s, 3H). ^13^C NMR (75 MHz, CD_3_COCD_3_): *δ* 160.7, 153.3, 141.4, 134.1, 130.8, 129.3, 129.0, 128.7, 122.4, 118.5, 117.0, 114.1, 73.7, 54.7.

2-(4-Methylphenyl)-3-nitro-2*H*-chromene (**5l**): Yellow solid. Yield: 40% (107 mg). ^1^H NMR (300 MHz, CD_3_COCD_3_): *δ* 8.30 (s, 1H), 7.60 (dd, *J* = 7.6, 1.7 Hz, 1H), 7.39 (ddd, *J* = 8.2, 7.4, 1.7 Hz, 1H), 7.36–7.30 (m, 2H), 7.21–7.14 (m, 2H), 7.07 (td, *J* = 7.5, 1.1 Hz, 1H), 6.86 (dt, *J* = 8.2, 1.1 Hz, 1H), 6.60 (s, 1H), 2.29 (s, 3H). ^13^C NMR (75 MHz, CD_3_COCD_3_): *δ* 153.4, 141.3, 139.4, 134.2, 134.1, 130.8, 129.4, 129.4, 127.1, 122.5, 118.5, 116.9, 73.9, 20.2.

2-(4-Fluorophenyl)-3-nitro-2*H*-chromene (**5m**): Yellow solid. Yield: 82% (222 mg). ^1^H NMR (300 MHz, CD_3_COCD_3_): *δ* 8.32 (s, 1H), 7.61 (dd, *J* = 7.5, 1.7 Hz, 1H), 7.57–7.47 (m, 2H), 7.41 (ddd, *J* = 8.3, 7.5, 1.7 Hz, 1H), 7.21–7.04 (m, 3H), 6.89 (dt, *J* = 8.3, 1.0 Hz, 1H), 6.66 (s, 1H). ^13^C NMR (75 MHz, CD_3_COCD_3_): *δ* 162.5, 153.1, 141.1, 134.3, 133.3, 131.0, 129.5, 122.7, 118.3, 116.9, 115.8, 115.5, 73.3.

2-(4-Chlorophenyl)-3-nitro-2*H*-chromene (**5n**): Yellow solid. Yield: 86% (247 mg). ^1^H NMR (300 MHz, CD_3_COCD_3_): *δ* 8.33 (s, 1H), 7.62 (dd, *J* = 7.6, 1.7 Hz, 1H), 7.56–7.36 (m, 5H), 7.10 (td, *J* = 7.6, 1.1 Hz, 1H), 6.95–6.85 (m, 1H), 6.66 (s, 1H). ^13^C NMR (75 MHz, CD_3_COCD_3_): *δ* 153.1, 140.9, 136.0, 134.8, 134.4, 131.0, 129.8, 129.0, 129.0, 122.8, 118.3, 116.9, 73.3.

2-(4-Bromophenyl)-3-nitro-2*H*-chromene (**5o**): Yellow solid. Yield: 95% (316 mg). ^1^H NMR (300 MHz, CD_3_COCD_3_): *δ* 8.32 (s, 1H), 7.61 (dd, *J* = 7.6, 1.7 Hz, 1H), 7.59–7.54 (m, 2H), 7.47–7.37 (m, 3H), 7.10 (td, *J* = 7.6, 1.1 Hz, 1H), 6.90 (dd, *J* = 8.2, 1.1 Hz, 1H), 6.65 (s, 1H). ^13^C NMR (75 MHz, CD_3_COCD_3_): *δ* 153.1, 140.8, 136.4, 134.4, 132.0, 131.0, 129.8, 129.3, 123.0, 122.8, 118.3, 116.9, 73.3.

6-Bromo-2-(4-bromophenyl)-3-nitro-2*H*-chromene (**5p**): Yellow solid. Yield: 71% (292 mg). ^1^H NMR (300 MHz, CD_3_COCD_3_) *δ* 8.32 (s, 1H), 7.82 (d, *J* = 2.5 Hz, 1H), 7.63–7.56 (m, 2H), 7.54 (dd, J = 8.7, 2.5 Hz, 1H), 7.47–7.41 (m, 2H), 6.87 (d, *J* = 8.7, 1H), 6.68 (s, 1H). ^13^C NMR (75 MHz, CD_3_COCD_3_) *δ* 152.2, 141.6, 136.5, 135.9, 132.9, 132.1, 129.3, 128.5, 123.3, 120.4, 119.0, 114.0, 73.6.

6-Chloro-2-(4-bromophenyl)-3-nitro-2*H*-chromene (**5q**): Yellow solid. Yield: 70% (257 mg). ^1^H NMR (300 MHz, CD_3_COCD_3_): *δ* 8.32 (s, 1H), 7.69 (d, *J* = 2.6 Hz, 1H), 7.62–7.55 (m, 2H), 7.48–7.43 (m, 2H), 7.41 (dd, *J* = 8.7, 2.6 Hz, 1H), 6.93 (d, *J* = 8.7, 1H), 6.68 (s, 1H). ^13^C NMR (75 MHz, CD_3_COCD_3_): *δ* 151.7, 141.8, 135.9, 133.6, 132.1, 130.0, 129.3, 128.6, 126.9, 123.3, 119.9, 118.6, 73.6.

6,8-Dibromo-2-(4-bromophenyl)- 3-nitro-2*H*-chromene (**5r**): Yellow solid. Yield: 44% (216 mg). ^1^H NMR (300 MHz, CDCl_3_) δ 6.68 (s, 1H, CH), 7.26 (d, *J* 8.6 Hz, 2H, 2 x CH), 7.42 (d, *J* 2.4 Hz, 1H, CH), 7.49 (d, *J* 8.5 Hz, 2H, 2 x CH), 7.68 (d, *J* 2.3 Hz, 1H, CH), 7.96 (s, 1H, CH) ppm. ^13^C NMR (75 MHz, CDCl_3_) δ 73.9, 112.4, 114.9, 120.6, 124.1, 127.5, 128.4, 131.5, 132.2, 134.6, 139.2, 142.3, 149.2.

8-Bromo-2-(4-bromophenyl)-6-chloro-3-nitro-2*H*-chromene (**5s**): Yellow solid. Yield: 51% (227 mg). m.p. 162.4–162.9 °C. HRMS (ESI+) [M]^+^ calcd. for C_15_H_8_Br_2_ClNO_3_, 442.8576; found, 442.8570. ^1^H NMR (300 MHz, CDCl_3_) δ 7.95 (s, 1H, CH), 7.53 (d, *J* = 2.4 Hz, 1H, CH), 7.44–7.50 (m, 2H, 2 x CH), 7.26–7.28 (m, 1H, CH), 7.24 (d, *J* = 8.3 Hz, 2H, 2 x CH), 6.66 (s, 1H, CH) ppm. ^13^C NMR (75 MHz, CDCl_3_) δ 148.7, 142.3, 136.5, 134.6, 132.2, 128.7, 128.4, 128.1, 127.7, 124.1, 120.1, 112.1, 73.9 ppm.

2-(4-Bromophenyl)-6,8-dichloro-3-nitro-2*H*-chromene (**5t**): Yellow solid. Yield: 47% (188 mg). m.p. 157.7–158.1 °C. HRMS (ESI+) [M]^+^ calcd. for C_15_H_8_BrCl_2_NO_3_, 398.9083; found, 398.9078. ^1^H NMR (300 MHz, CDCl_3_) δ 7.95 (s, 1H, CH), 7.47 (d, *J* = 8.6 Hz, 2H, 2 x CH), 7.37 (d, *J* = 2.4 Hz, 1H, CH), 7.23 (s, 1H, CH), 7.23 (d, *J* = 8.1 Hz, 2H, 2 x CH), 6.65 (s, 1H, CH) ppm. ^13^C NMR (75 MHz, CDCl_3_) δ 147.7, 142.4, 133.7, 134.7, 132.2, 128.4, 127.9, 127.7, 127.6, 124.1, 123.6, 120.2, 73.9 ppm.

### 4.2. Biological Activity

#### 4.2.1. Bacterial Strains 

The synthetized 3-nitro-2*H*-chromenes **5** were tested against a panel of MDR strains of clinical origin. Methicillin-resistant *Staphylococcus aureus* (MRSA) and P. aeruginosa strains were isolated from cystic fibrosis patients at the Hospital Clinic of Barcelona (Barcelona, Spain). *Staphylococcus epidermidis* strains were obtained from wounds at the Hospital Clinic of Barcelona (Barcelona, Spain). *A. baumannii* strains were isolated from cerebrospinal fluid at the Hospital Virgen de Rocío (Seville, Spain). 

#### 4.2.2. Minimal Inhibitory Concentration Determination

The minimum inhibitory concentrations (MICs) were determined in triplicate by the broth microdilution in 96-well round bottom microtiter plates following the Clinical & Laboratory Standards Institute (CLSI) guidelines [70]. Bacteria were cultured in ISO-Sensitest broth (Oxoid, Madrid, Spain) at 37 °C for 18 h, and then suspended in physiological saline (0.9%, *w/v* NaCl) to achieve a final density of bacterial inoculum of 5 × 10^5^ CFU/mL. The tested concentrations of gold ranged from 0.05 mg/L to 1024 mg/L, and MIC values were defined as the lowest concentration of the compound that inhibited visible growth.

#### 4.2.3. In Vitro Cytotoxicity Assay

Cytotoxicity in terms of antiproliferative effect was assessed using the XTT (sodium 2,3-bis(2-methoxy-4-nitro-5-sulfophenyl)-5-[(phenylamino)carbonyl]-2*H*-tetrazolium) colorimetric assay (XTT Cell Proliferation Assay Kit, Canvax Biotech, Córdoba, Spain). The cell line used in this study was A549 lung epithelial cells. Analyses were conducted in sterile 96-well microplates, and cells were spread to an initial concentration of 1 × 10^4^ cells/well and incubated at 37 °C in 5% CO_2_ for 24 h. After this time, 50 μL of XTT were added to each well, and the plates were gently shaken and incubated for an additional 4 h. The results were measured on the Epoch™ spectrophotometer plate reader (Agilent, Santa Clara, CA, USA) at a wavelength of 450–500 nm and 630–690 nm. 

#### 4.2.4. Statistical Analysis

For data analysis, the statistical software R commander 4.1. was used, whereas Graphics were produced employing GraphPad Prism 9 software.

## 5. Conclusions

To sum up, 20 nitrochromene derivatives were synthesized, and their antibacterial activity against panel Gram-positive and Gram-negative bacterial cell lines was assessed. Halogenated nitrochromenes were found to possess antibacterial activity against multidrug-resistant clinical isolates of Gram-positive *S. aureus* and *S. epidermidis*. It is noteworthy that the introduction of additional halogen atoms in the 3-nitro-2*H*-chromene core motif has significant influence on the antimicrobial activity. Thus, tri-halogenated displayed the highest antibacterial activity, much better than the pharmacological reference antibiotic ciprofloxacin. Among them, 2-(4-bromophenyl)-6-bromo-8-chloro-3-nitro-2*H*-chromene 5s displays an optimal therapeutic index and has successfully passed the stage of in silico testing of ADME properties; therefore, it could be a good lead for further research.

In conclusion, halogenated nitrochromenes stand out as potential leads in the development of effective antibacterial drugs against multidrug-resistant strains of *S. aureus* and *S. epidermidis*, which are important pathogens related to nosocomial infections.

## Figures and Tables

**Table 1:** Synthesis of the target 3-nitro-2*H*-chromenes **5**.

“Synthesis Route”						
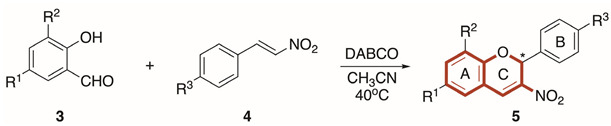						
Aldehyde	R^1^	R^2^	Nitrostyrene	R^3^	Chromene	Yield
**3a**	H	H	**4a**	H	**5a**	60
**3b**	OMe	H	**4a**	H	**5b**	67
**3c**	Me	H	**4a**	H	**5c**	63
**3d**	F	H	**4a**	H	**5d**	50
**3e**	Cl	H	**4a**	H	**5e**	40
**3f**	Br	H	**4a**	H	**5f**	50
**3g**	OCF_3_	H	**4a**	H	**5g**	59
**3h**	NO_2_	H	**4a**	H	-	-
**3i**	H	OMe	**4a**	H	**5h**	65
**3j**	H	Me	**4a**	H	**5i**	52
**3k**	H	OEt	**4a**	H	**5j**	66
**3l**	*^t^*Bu	*^t^*Bu	**4a**	H	-	-
**3a**	H	H	**4b**	OMe	**5k**	71
**3a**	H	H	**4c**	Me	**5l**	40
**3a**	H	H	**4d**	F	**5m**	82
**3a**	H	H	**4e**	Cl	**5n**	86
**3a**	H	H	**4f**	Br	**5o**	95
**3f**	Br	H	**4f**	Br	**5p**	71
**3e**	Cl	H	**4f**	Br	**5q**	70
**3m**	Br	Br	**4f**	Br	**5r**	44
**3n**	Br	Cl	**4f**	Br	**5s**	51
**3o**	Cl	Cl	**4f**	Br	**5t**	47

**Table 2:** Antibacterial activity of 3-nitro-2*H*-chromenes **5**.

5	Antimicrobial Activity (μg/mL)				
	MRSA	*S. epidermidis*	*A. baumannii*	*P. aeruginosa*	*E. coli*
**5a**	32–64	32–64	>128	>128	>128
**5b**	>128	>128	>128	>128	>128
**5c**	>128	>128	>128	>128	>128
**5d**	32	32	>128	>128	>128
**5e**	16	16	>128	>128	>128
**5f**	16	16	>128	>128	>128
**5g**	16	16	>128	>128	>128
**5h**	128	128	>128	>128	>128
**5i**	128	128	>128	>128	>128
**5j**	>128	>128	>128	>128	>128
**5k**	>128	>128	>128	>128	>128
**5l**	16	>128	>128	>128	>128
**5m**	32	32	>128	>128	>128
**5n**	8	16	>128	>128	>128
**5o**	8	16	>128	>128	>128
**5p**	16	2–8	>128	>128	>128
**5q**	8	4–8	>128	>128	>128
**5r**	4	4	>128	>128	>128
**5s**	2–4	1–4	>128	>128	>128
**5t**	4	2–4	>128	>128	>128
CIP ^a^	>128	>128	>128	>128	>128
TOB ^b^	2–128	0.25–32	64–128	32–128	>128

^a^ Ciprofloxacin. ^b^ Tobramycin.

## Data Availability

The original contributions presented in this study are included in the article/Supplementary Materials. Further inquiries can be directed to the corresponding authors.

## Supplementary Materials

The following supporting information can be downloaded at: https://www.mdpi.com/article/10.3390/antibiotics14030218/s1. Copies of the ^1^H and ^13^C NMR spectra for all the synthetized 3-nitro-2*H*-chromenes 5.
