# Supplementary material for: Halogenated 3-Nitro-2H-Chromenes as Potential Agents Against Multidrug-Resistant Bacteria
Source: Antibiotics (Basel). 2025 Feb 21;14(3):218. doi: 10.3390/antibiotics14030218 (PMC11939745; doi:10.3390/antibiotics14030218)
Supplement: Supplementary file 1 [file antibiotics-14-00218-s001.zip › antibiotics-3471034-supplementary.pdf]

3-Nitro-2*H*-chromenes as Potential Agents Against Multi-drug-Resistant Bacteria

Patrícia I. C. Godinho[1], Paula Pérez-Ramos[2], Yaiza Gabasa[3,4], Sara M. Soto[3,4], Raquel G. Soengas[2], Artur M. S. Silva[1]

[1] LAQV-REQUIMTE, Department of Chemistry, University of Aveiro, 3810-193 Aveiro, Portugal.
[2] Department of Organic and Inorganic Chemistry, University of Oviedo, and Instituto Universitario de Química Organometálica Enrique Moles, C/ Julián Clavería 8, 33006, Oviedo Spain.
[3] ISGlobal, Hospital Clínic—Universitat de Barcelona, 08036 Barcelona, Spain.
[4] CIBER Enfermedades Infecciosas (CIBERINFEC), Instituto de Salud Carlos III, Madrid, Spain

Correspondence: artur.silva@ua.pt; rsoengas@uniovi.es

NMR spectra of 3-nitro-2*H*-chromenes **5**……………………...…………………….………….......S2-S18

3-Nitro-2-phenyl-2*H*-chromene (5a)

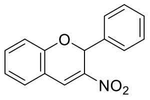

5a

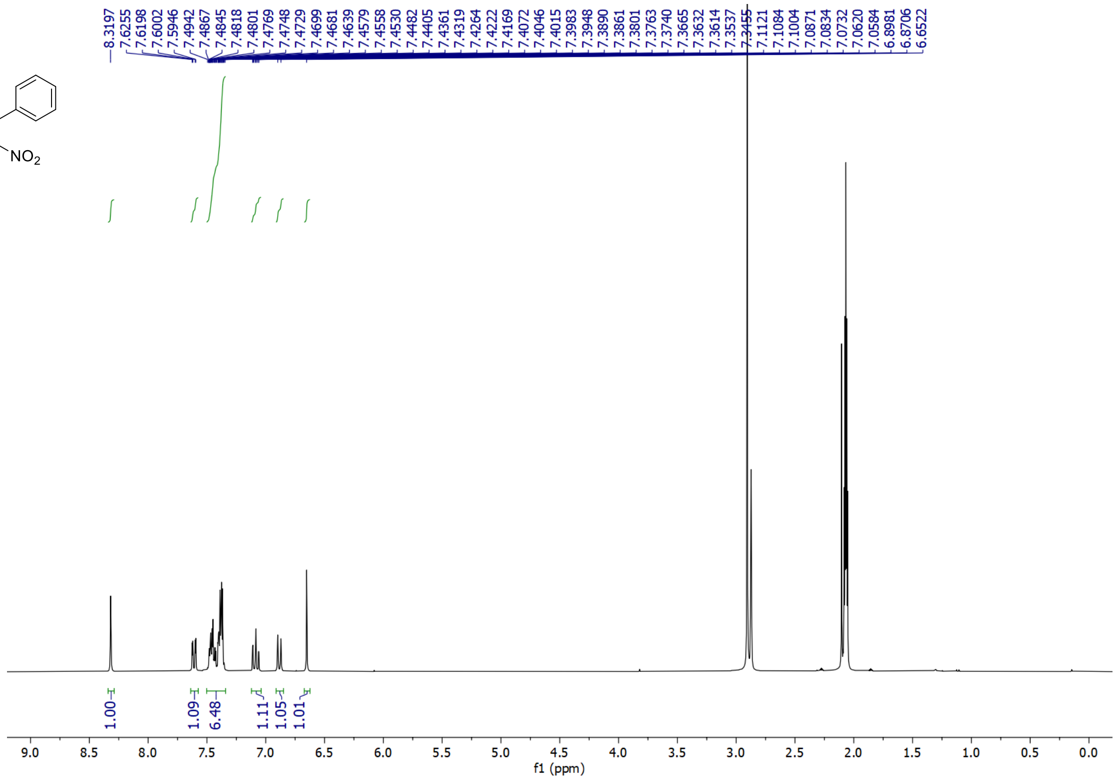

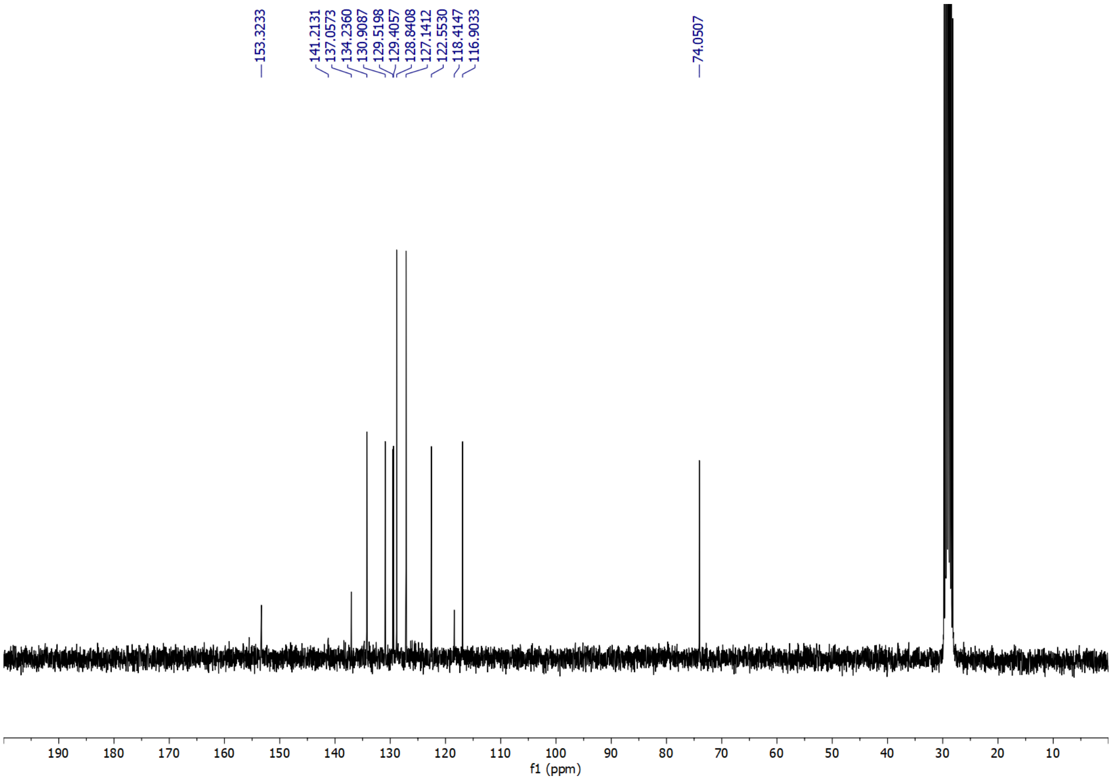

6-Methoxy-3-nitro-2-phenyl-2*H*-chromene (5b)

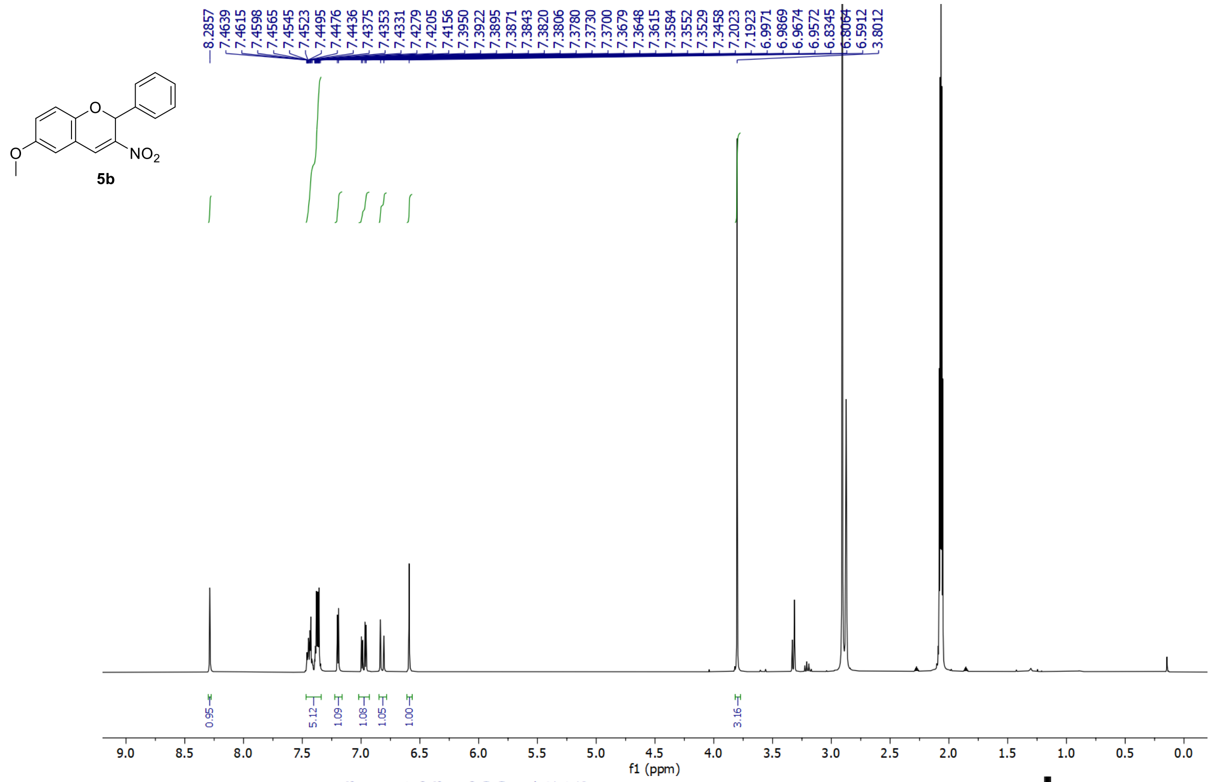

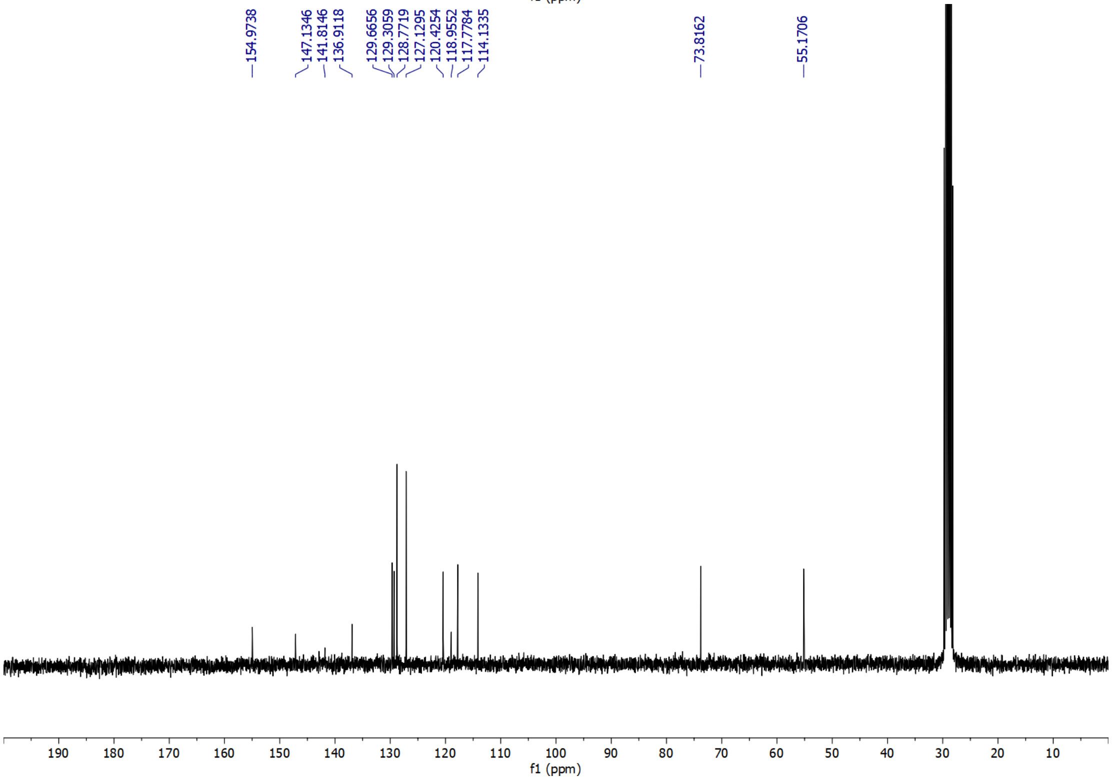

6-Methyl-3-nitro-2-phenyl-2H-chromene (5c)

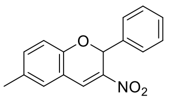
O
NO_2

5c

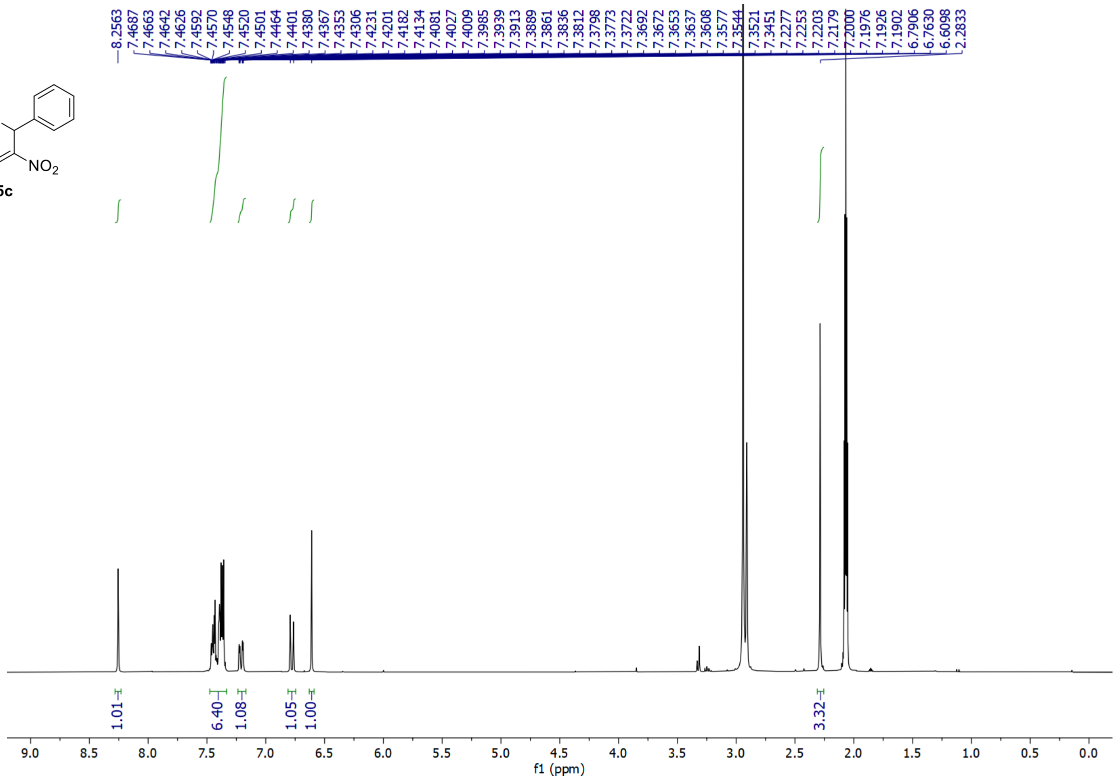
8.2563
7.4687
7.4663
7.4642
7.4626
7.4592
7.4570
7.4548
7.4520
7.4501
7.4464
7.4401
7.4380
7.4367
7.4353
7.4306
7.4231
7.4201
7.4182
7.4134
7.4081
7.4027
7.4009
7.3985
7.3939
7.3913
7.3889
7.3861
7.3836
7.3812
7.3798
7.3773
7.3722
7.3692
7.3672
7.3653
7.3637
7.3608
7.3577
7.3544
7.3521
7.3451
7.2277
7.2253
7.2203
7.2179
7.2000
7.1976
7.1926
7.1902
6.7906
6.7630
6.6098
2.2833
1.01
6.40
1.08
1.05
1.00
3.32
9.0 8.5 8.0 7.5 7.0 6.5 6.0 5.5 5.0 4.5 4.0 3.5 3.0 2.5 2.0 1.5 1.0 0.5 0.0
f1 (ppm)

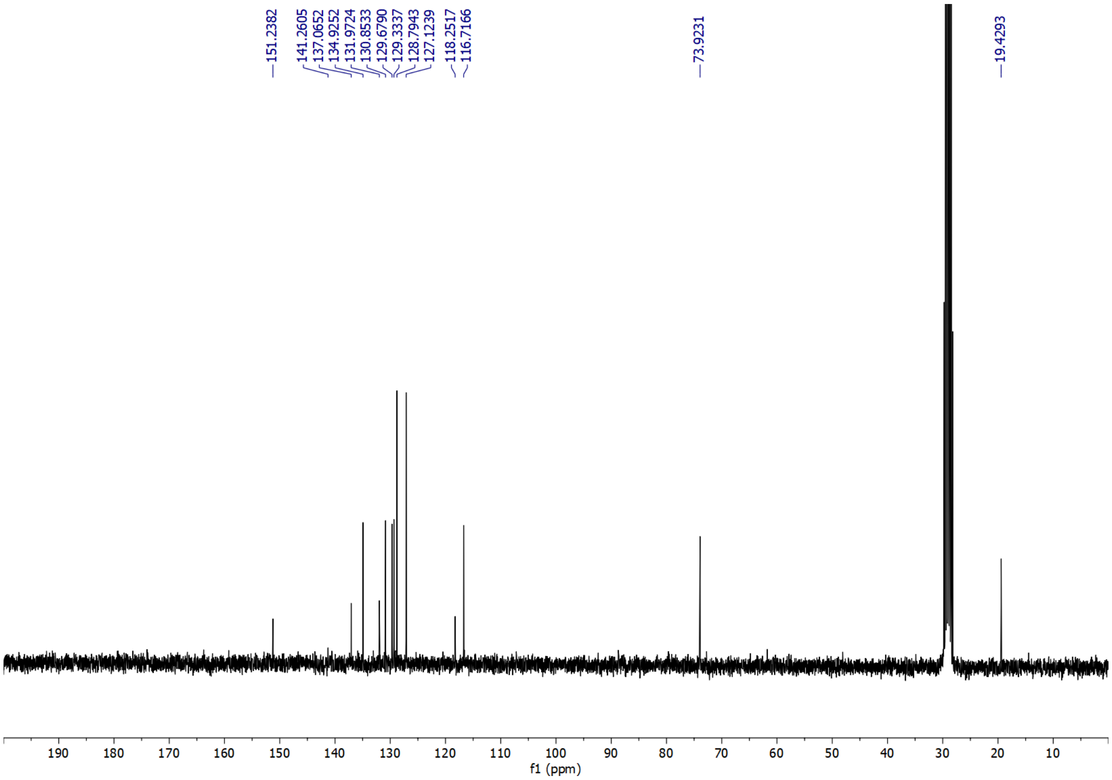
151.2382
141.2605
137.0652
134.9252
131.9724
130.8533
129.6790
129.3337
128.7943
127.1239
118.2517
116.7166
73.9231
19.4293
190 180 170 160 150 140 130 120 110 100 90 80 70 60 50 40 30 20 10
f1 (ppm)

6-Fluoro-3-nitro-2-phenyl-2H-chromene (5d)

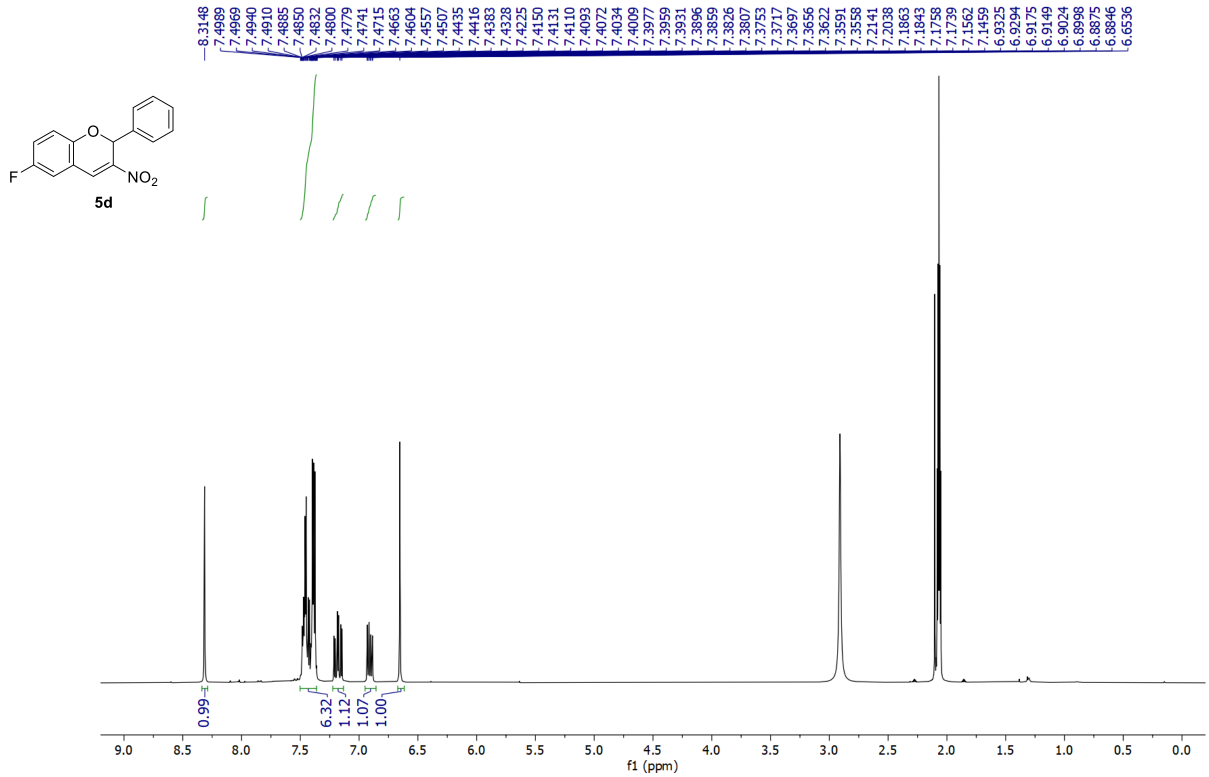

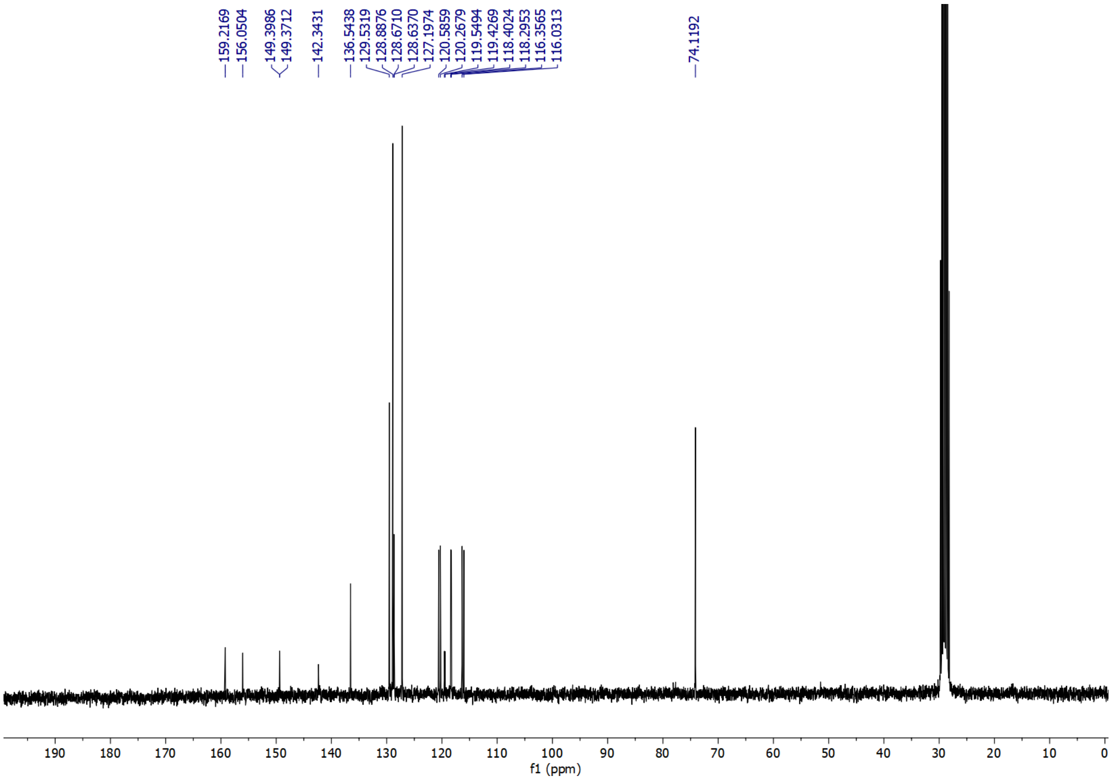

6-Chloro-3-nitro-2-phenyl-2H-chromene (5e)

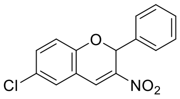
O
Cl
NO_2

5e

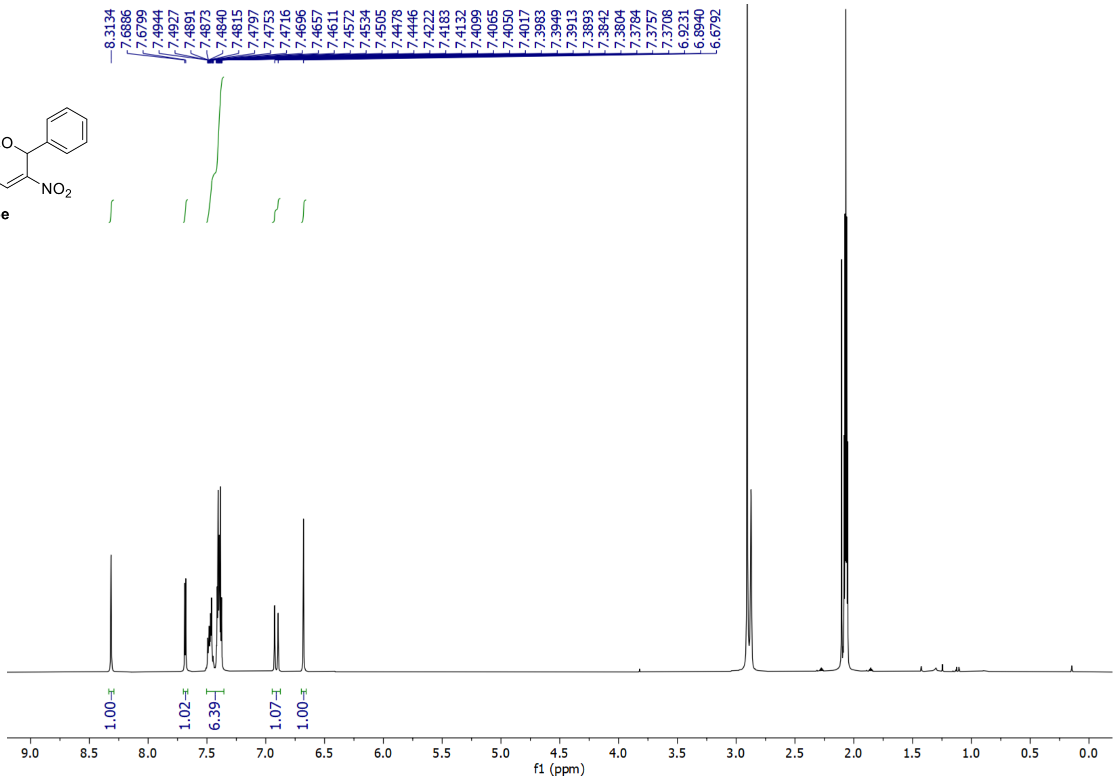
8.3134
7.6886
7.6799
7.4944
7.4927
7.4891
7.4873
7.4840
7.4815
7.4797
7.4753
7.4716
7.4696
7.4657
7.4611
7.4572
7.4534
7.4505
7.4478
7.4446
7.4222
7.4183
7.4132
7.4099
7.4065
7.4050
7.4017
7.3983
7.3949
7.3913
7.3893
7.3842
7.3804
7.3784
7.3757
7.3708
6.9231
6.8940
6.6792
1.00
1.02
6.39
1.07
1.00
9.0
8.5
8.0
7.5
7.0
6.5
6.0
5.5
5.0
4.5
4.0
3.5
3.0
2.5
2.0
1.5
1.0
0.5
0.0
f1 (ppm)

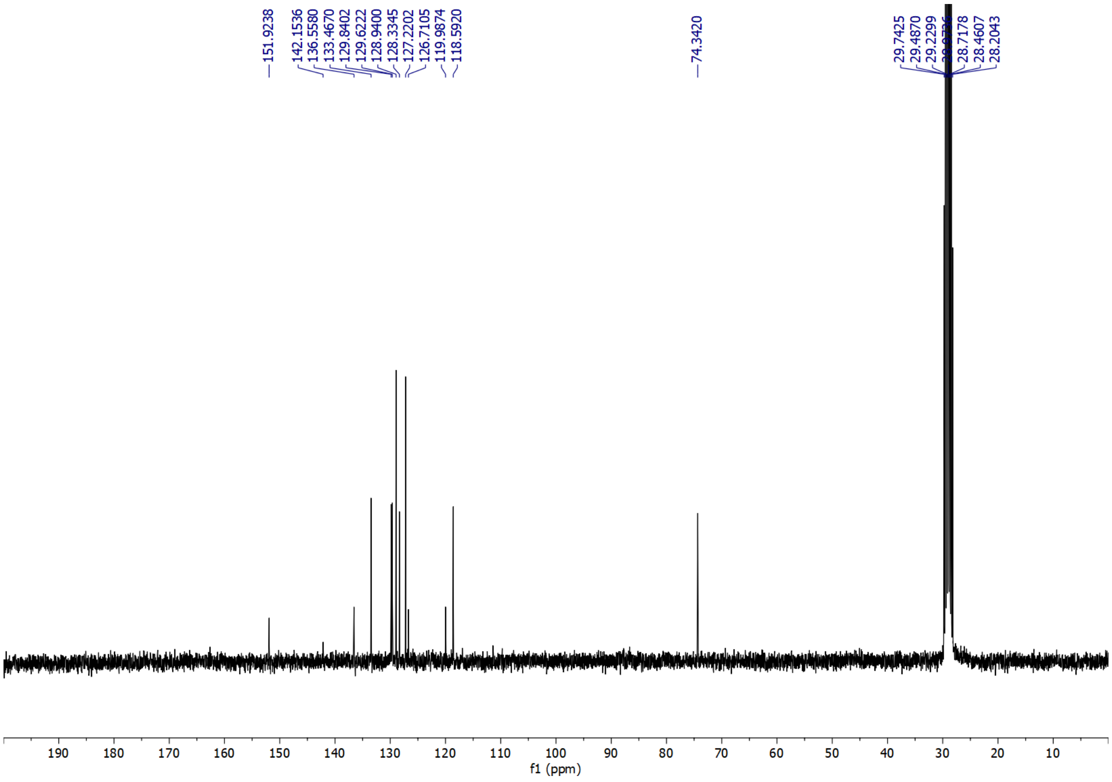
151.9238
142.1536
136.5580
133.4670
129.8402
129.6222
128.9400
128.3345
127.2202
126.7105
119.9874
118.5920
74.3420
29.7425
29.4870
29.2299
28.7178
28.4607
28.2043
190
180
170
160
150
140
130
120
110
100
90
80
70
60
50
40
30
20
10
f1 (ppm)

6-Bromo-3-nitro-2-phenyl-2*H*-chromene (5f)

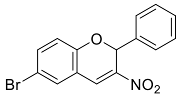

5f

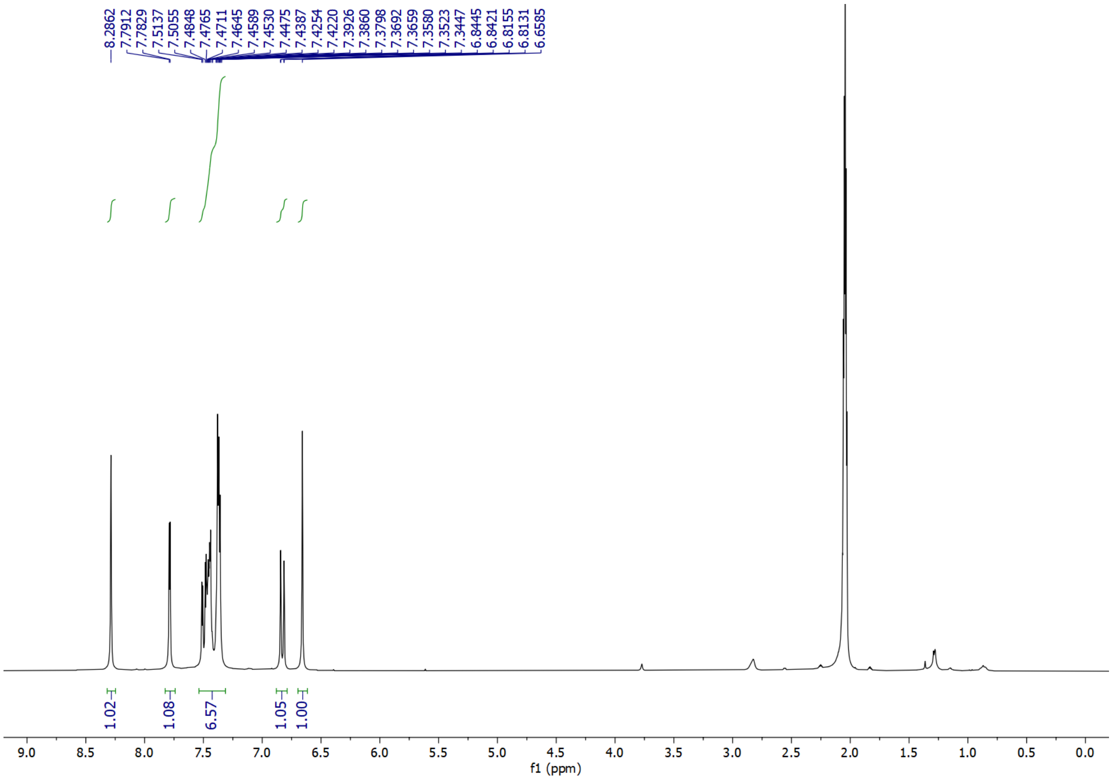

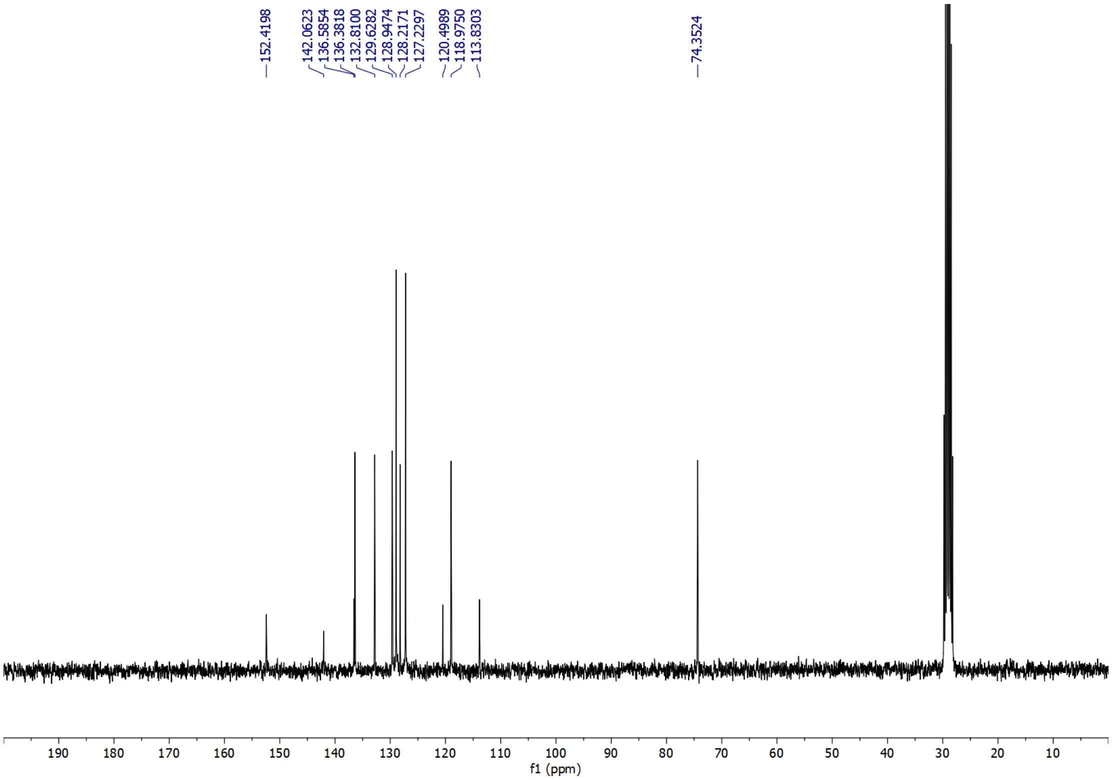

6-Trifluoromethoxy-3-nitro-2-phenyl-2*H*-chromene (5g)

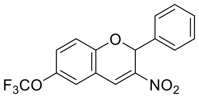

5g

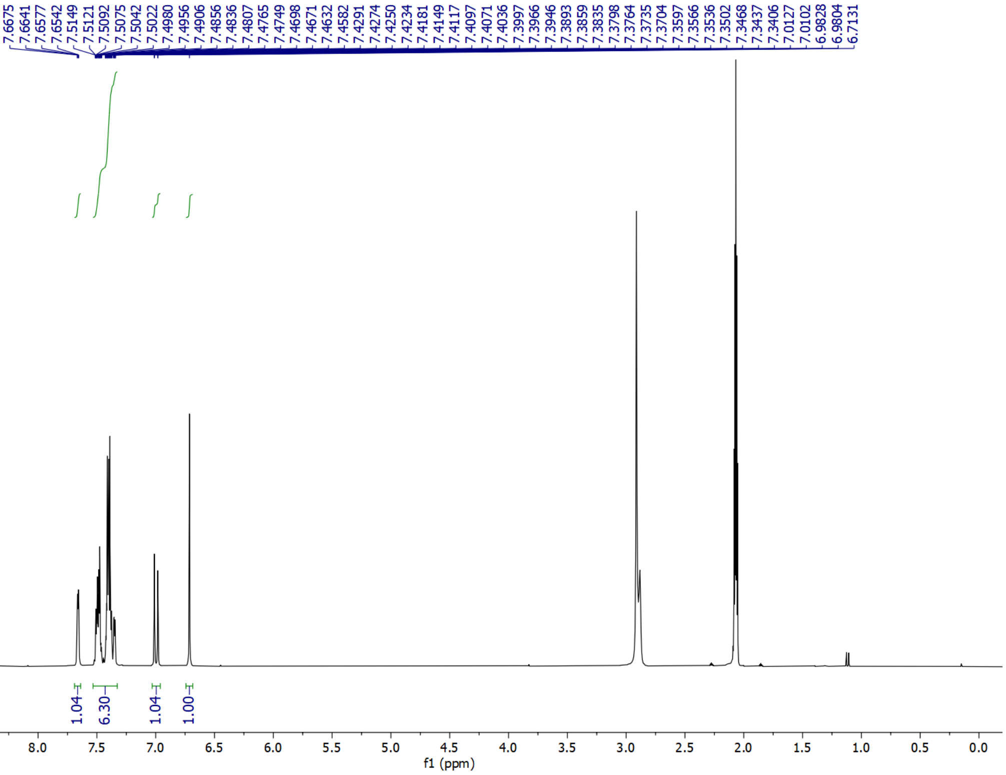

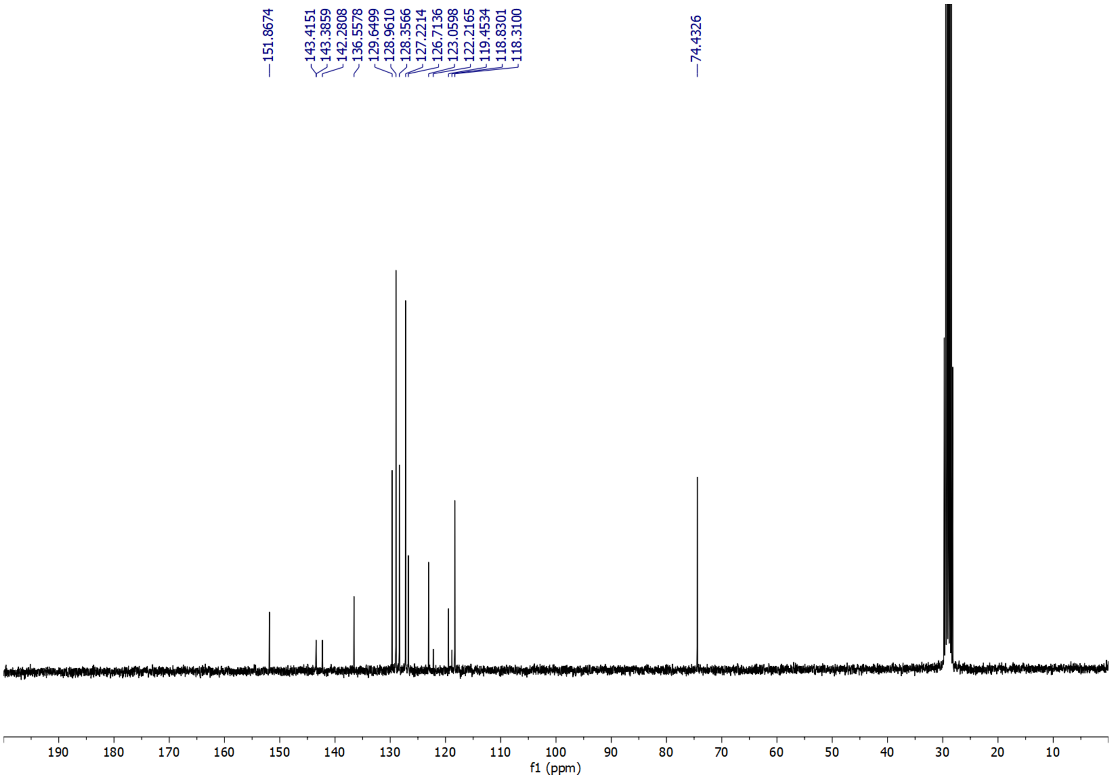

8-Methoxy-3-nitro-2-phenyl-2*H*-chromene (5h)

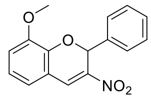

5h

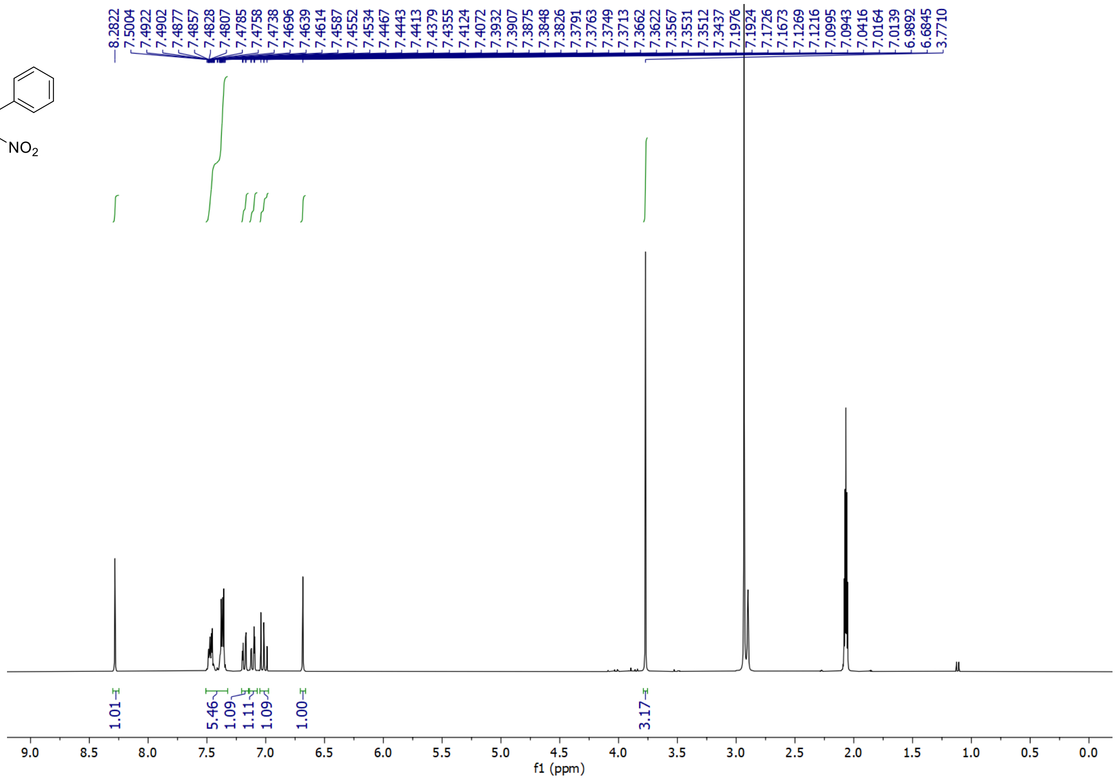

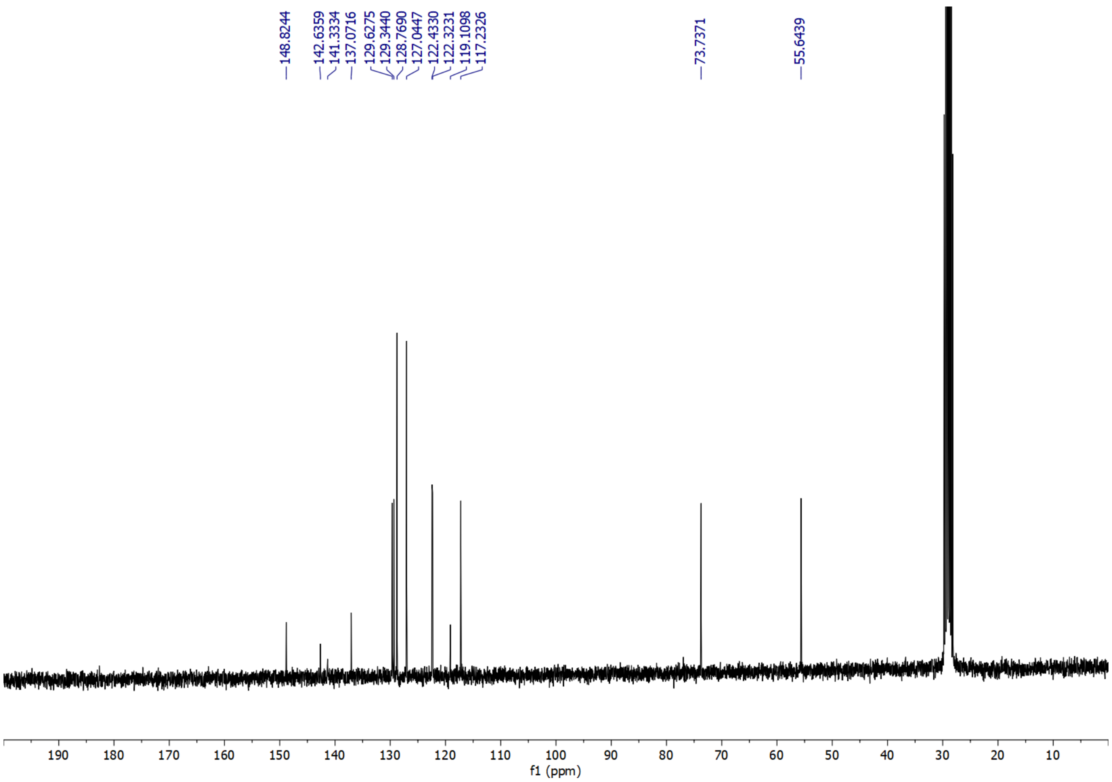

8-Methyl-3-nitro-2-phenyl-2*H*-chromene (5i)

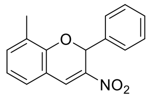

5i

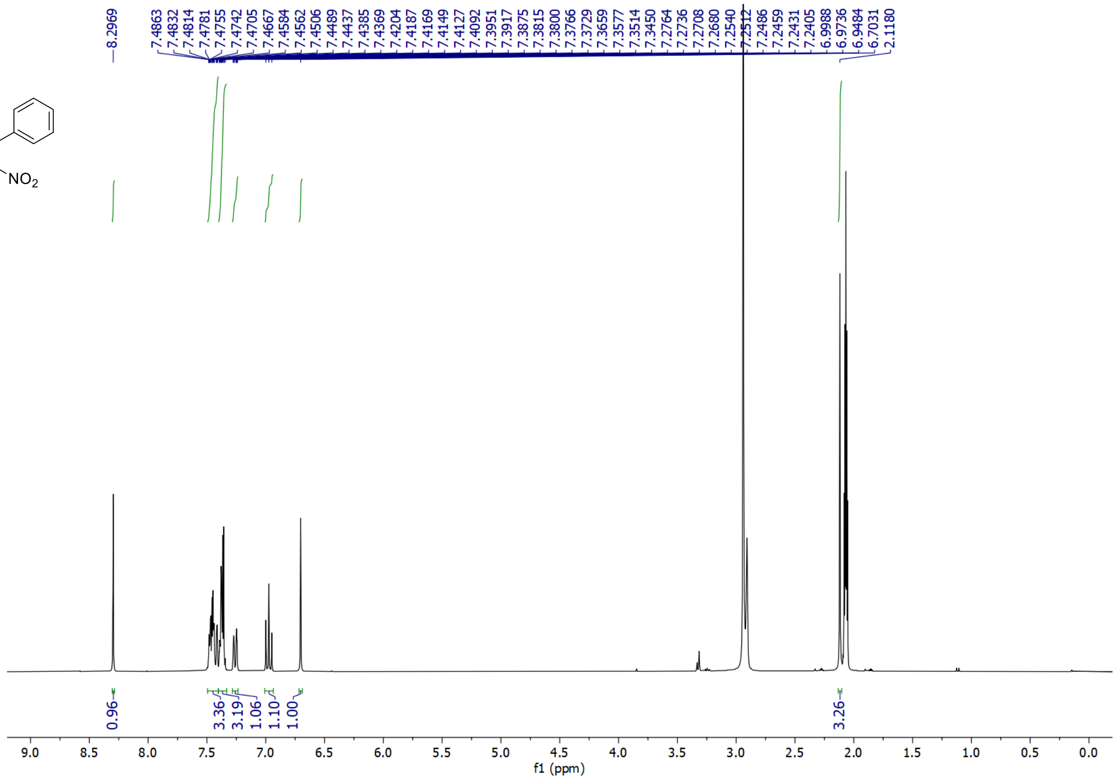

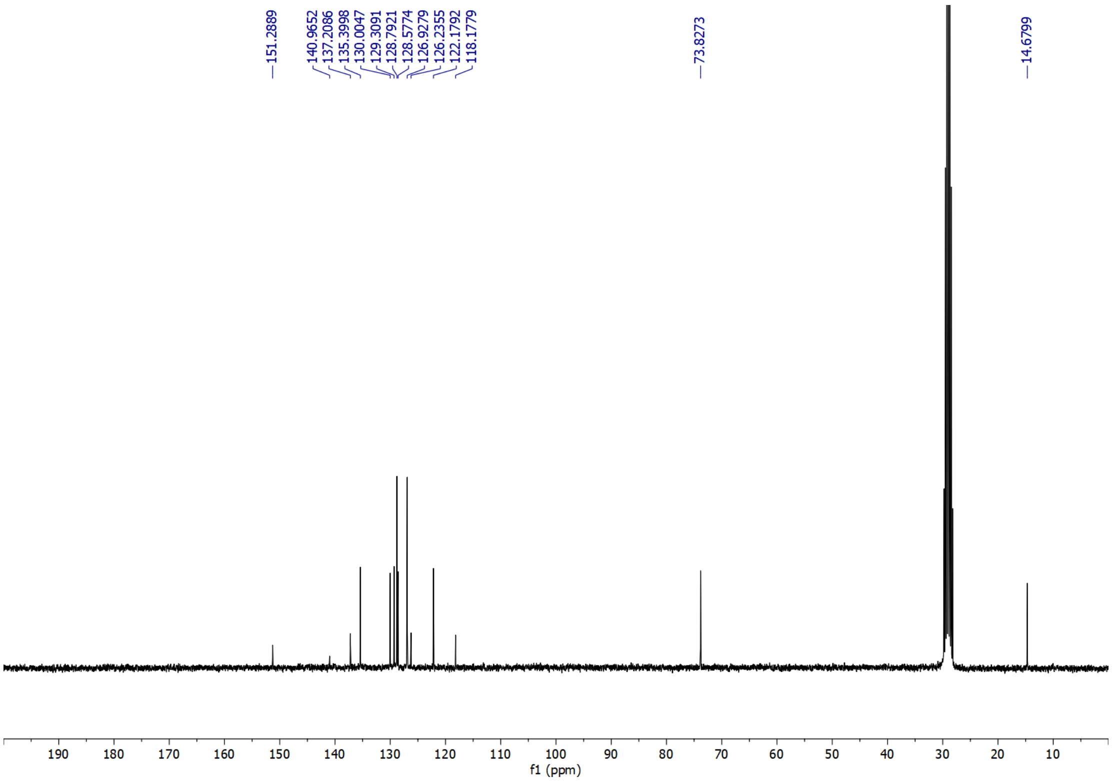

8-Ethoxy-3-nitro-2-phenyl-2*H*-chromene (5j)

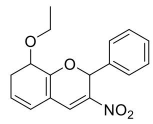

5j

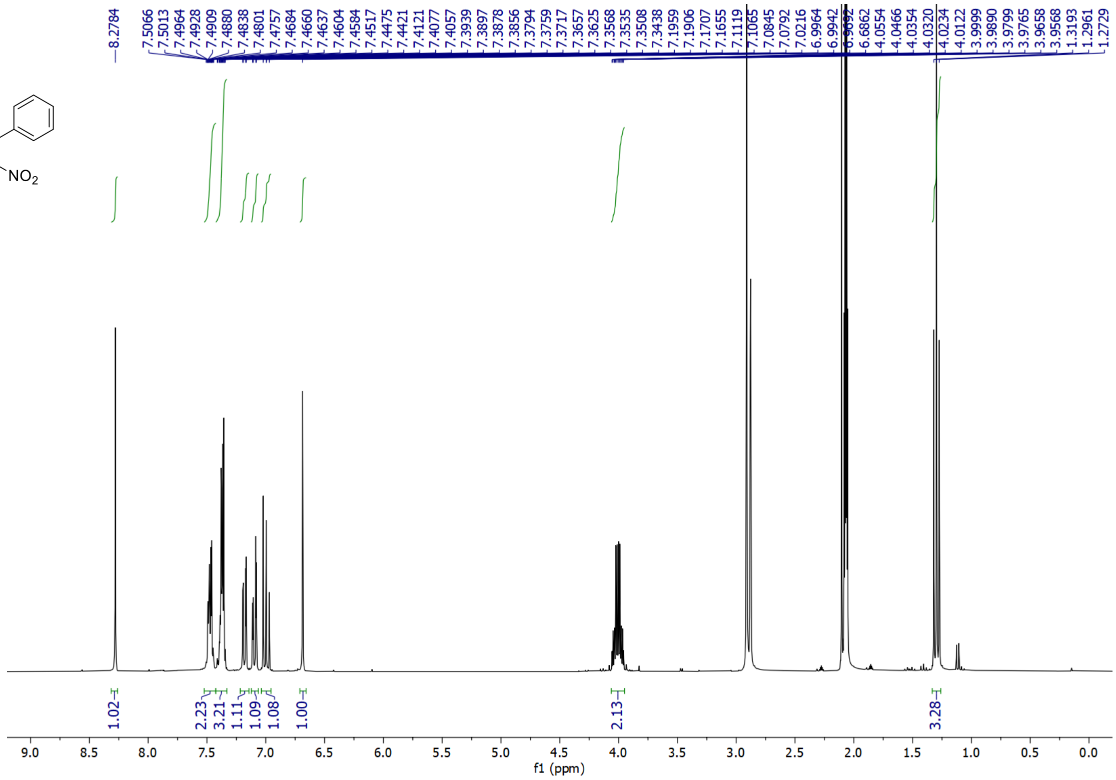

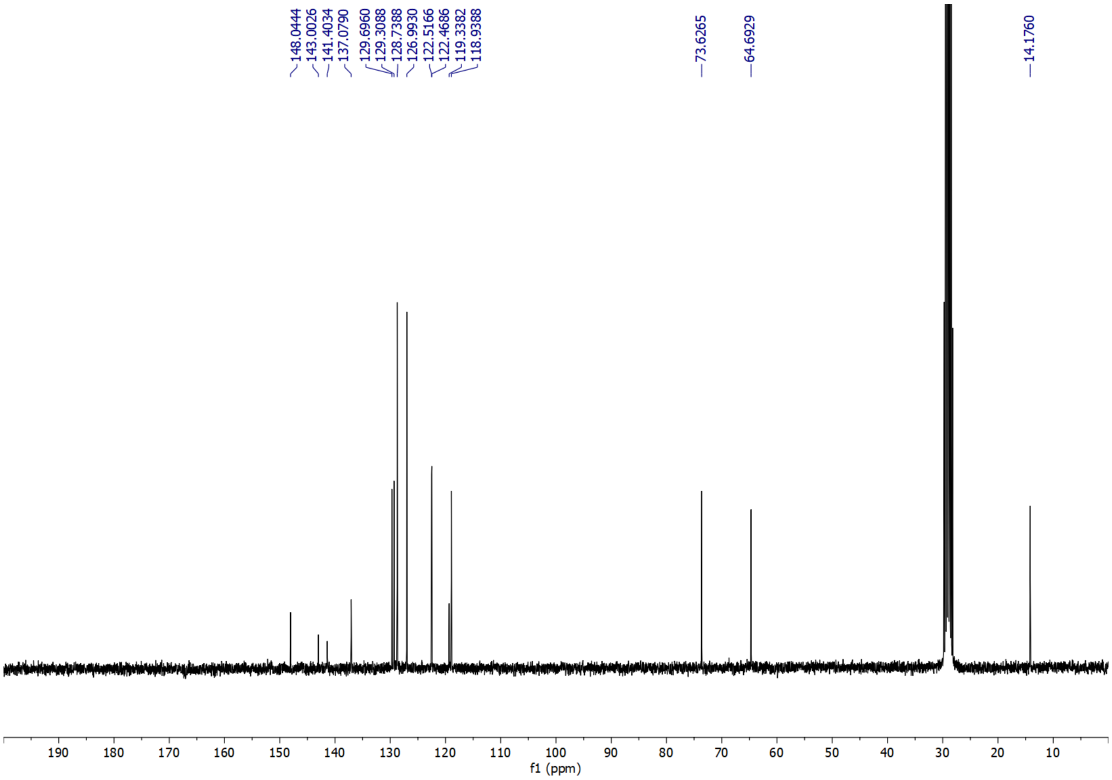

2-(4-Methoxyphenyl)-3-nitro-2*H*-chromene (5k)

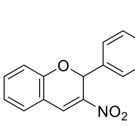

5k

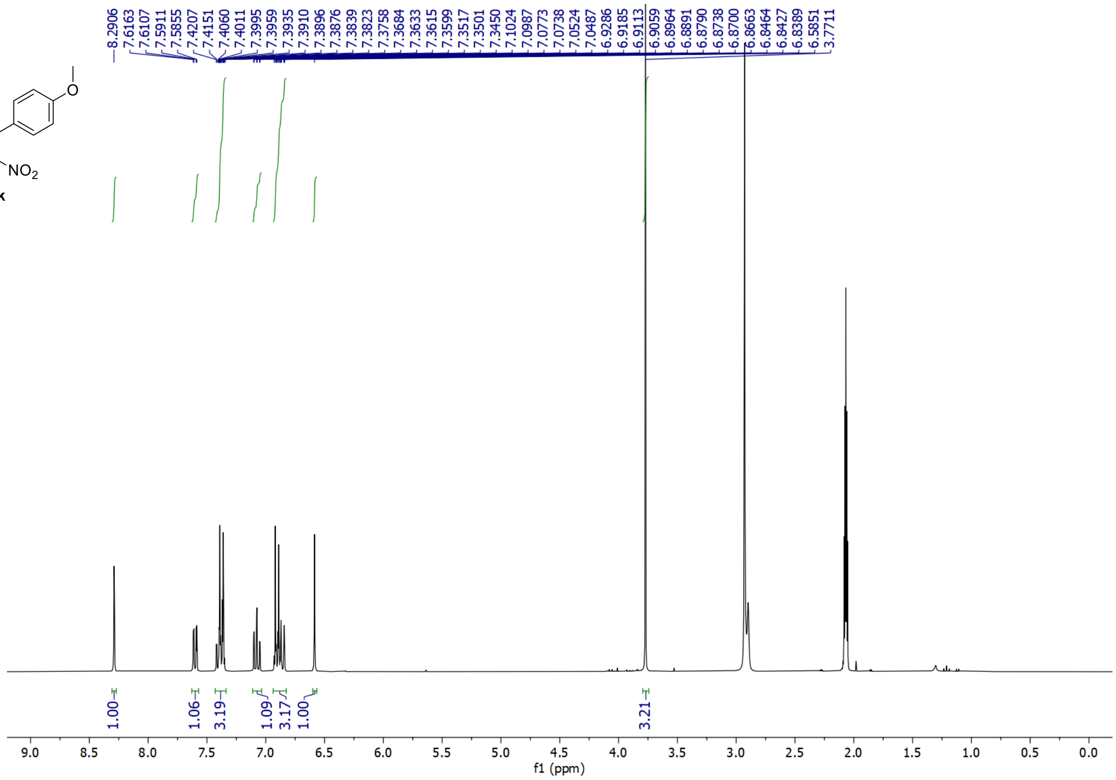

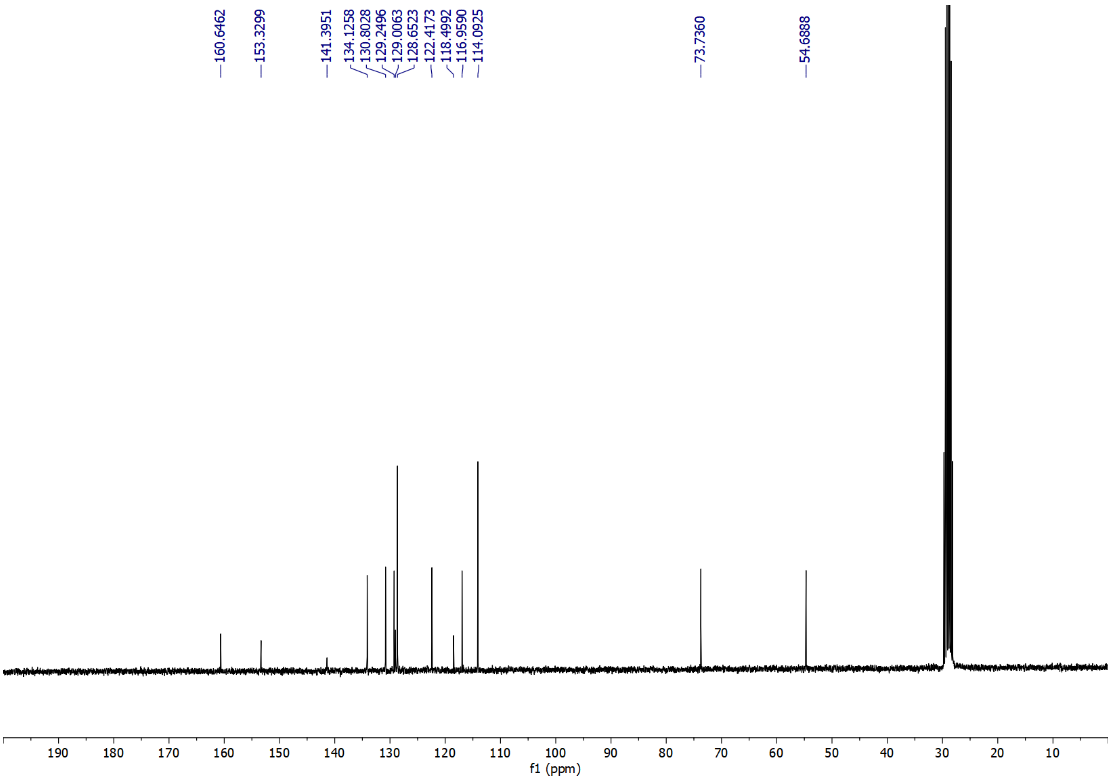

2-(4-Methylphenyl)-3-nitro-2*H*-chromene (5l)

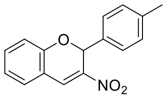

5l

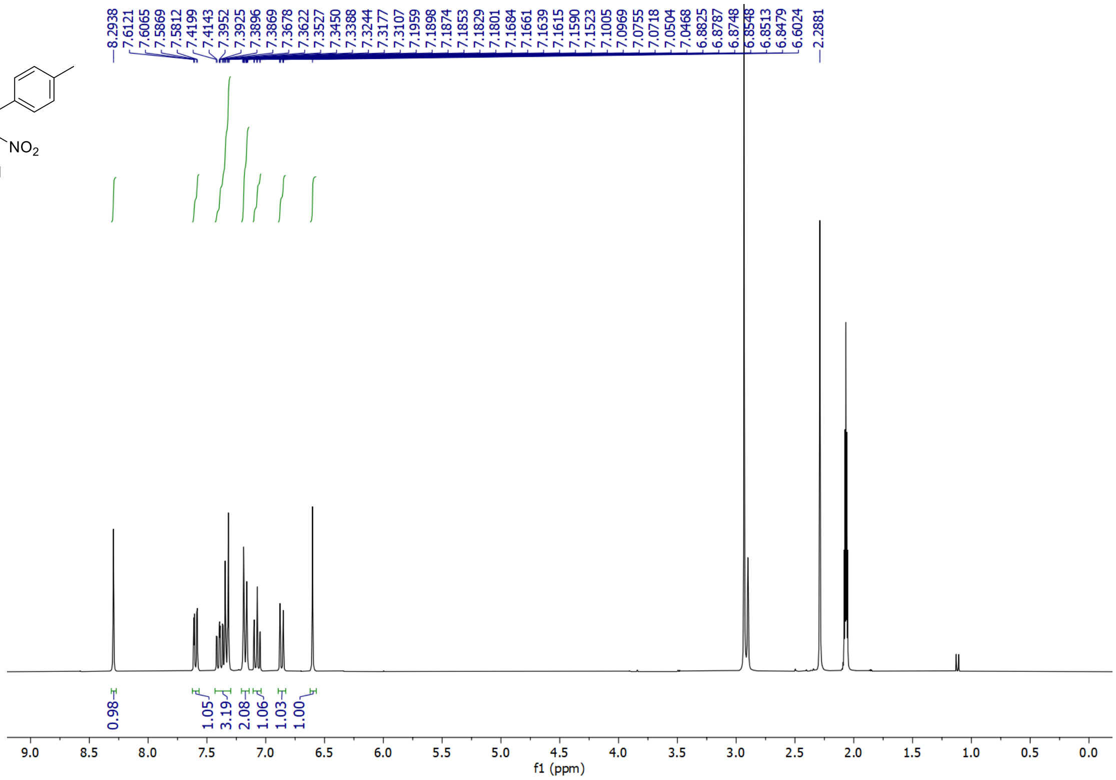

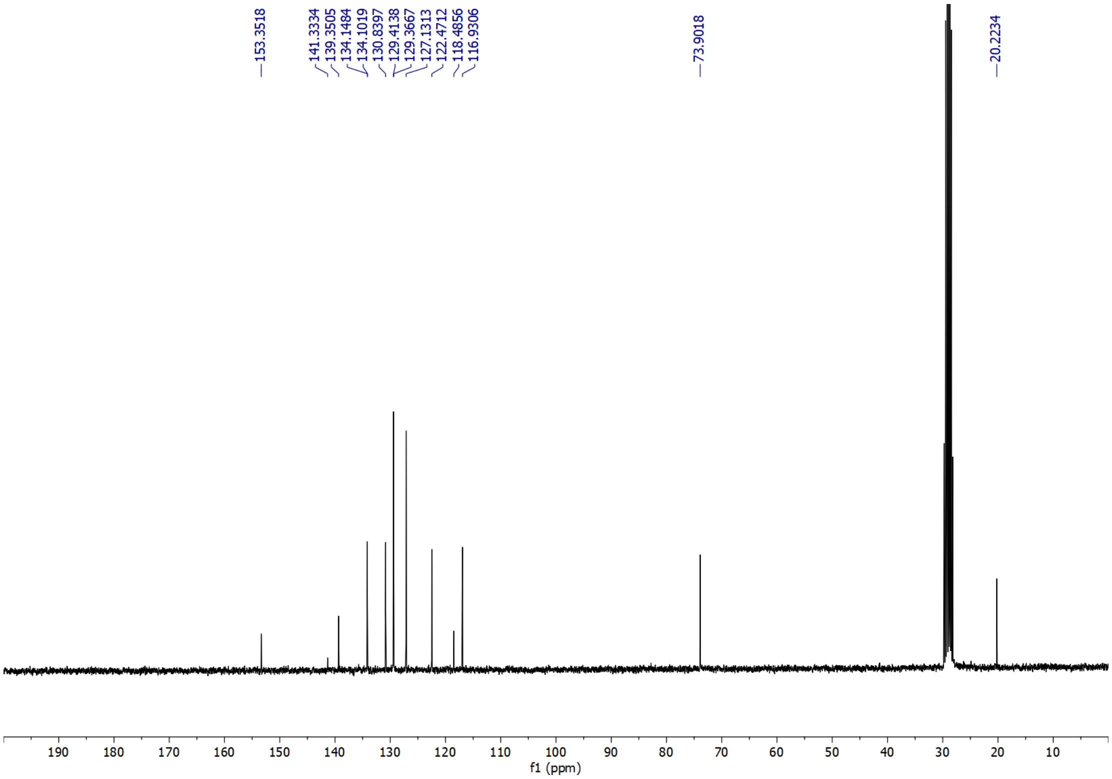

2-(4-Fluorophenyl)-3-nitro-2*H*-chromene (5m)

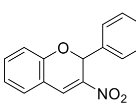

5m

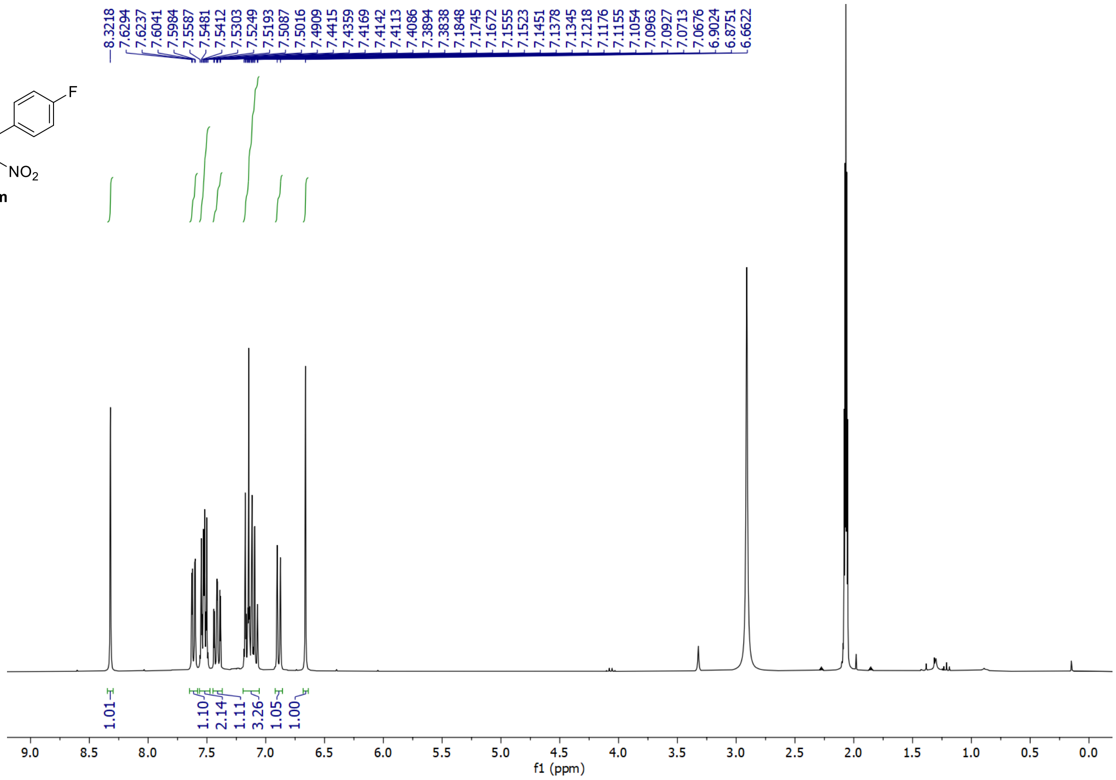

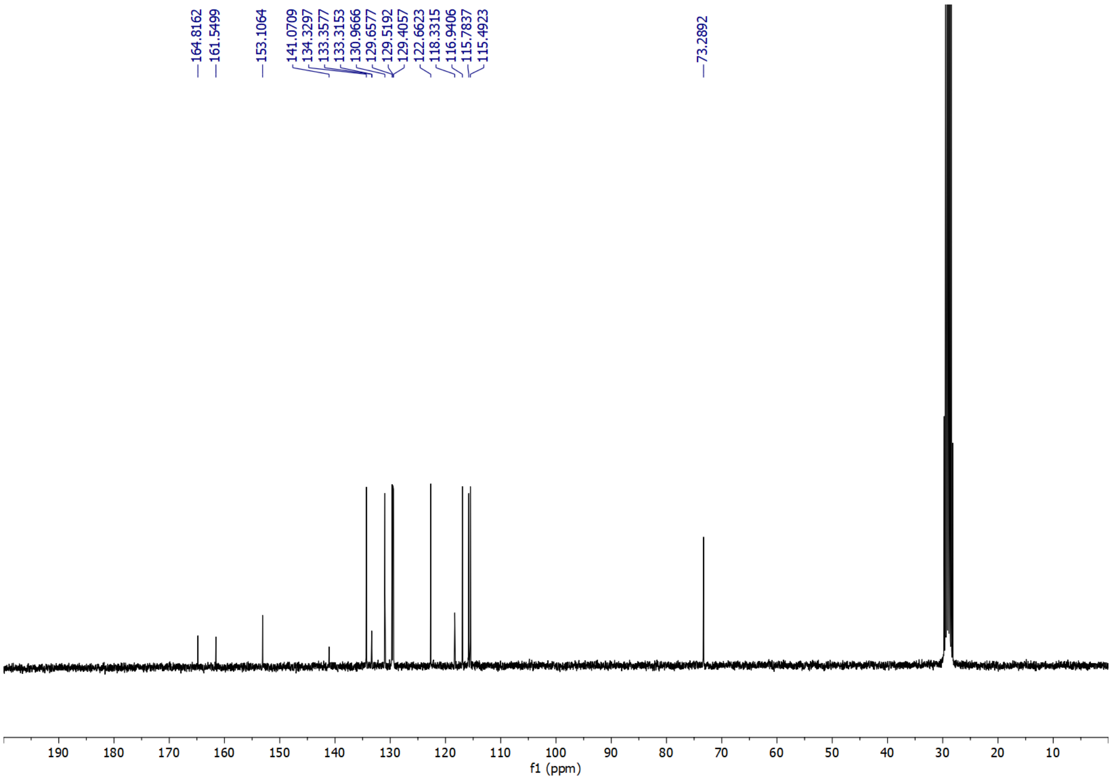

2-(4-Chlorophenyl)-3-nitro-2*H*-chromene (5n)

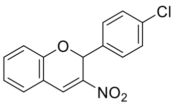

5n

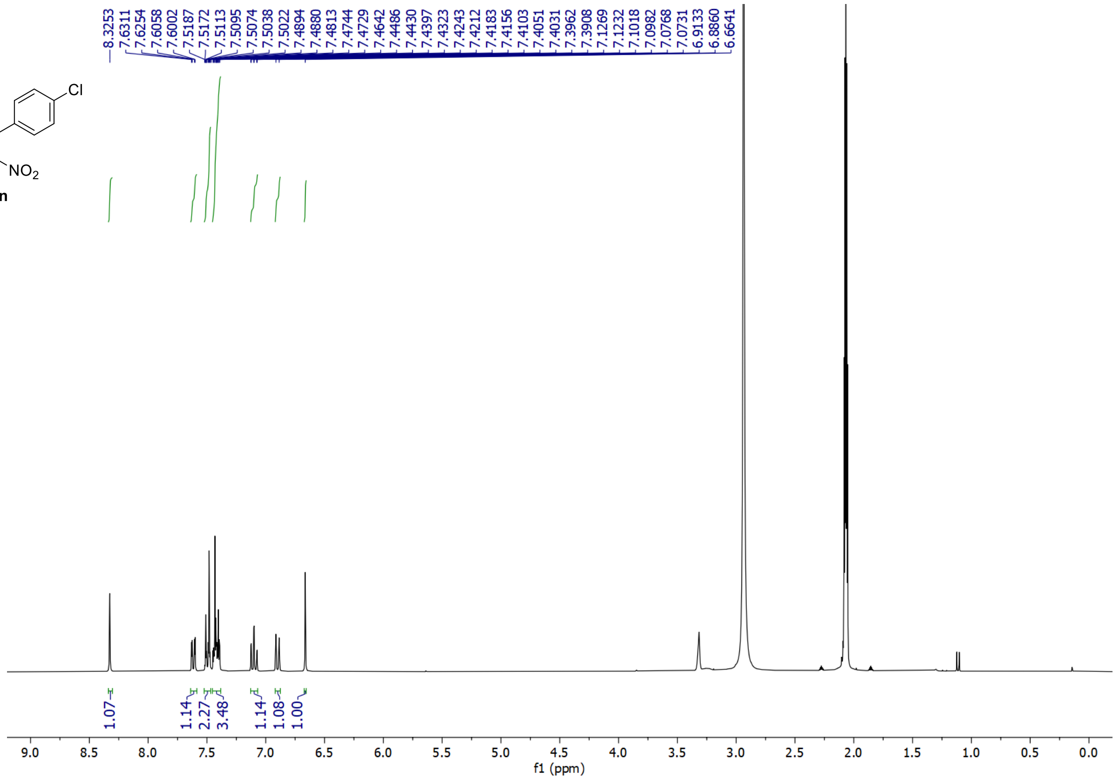

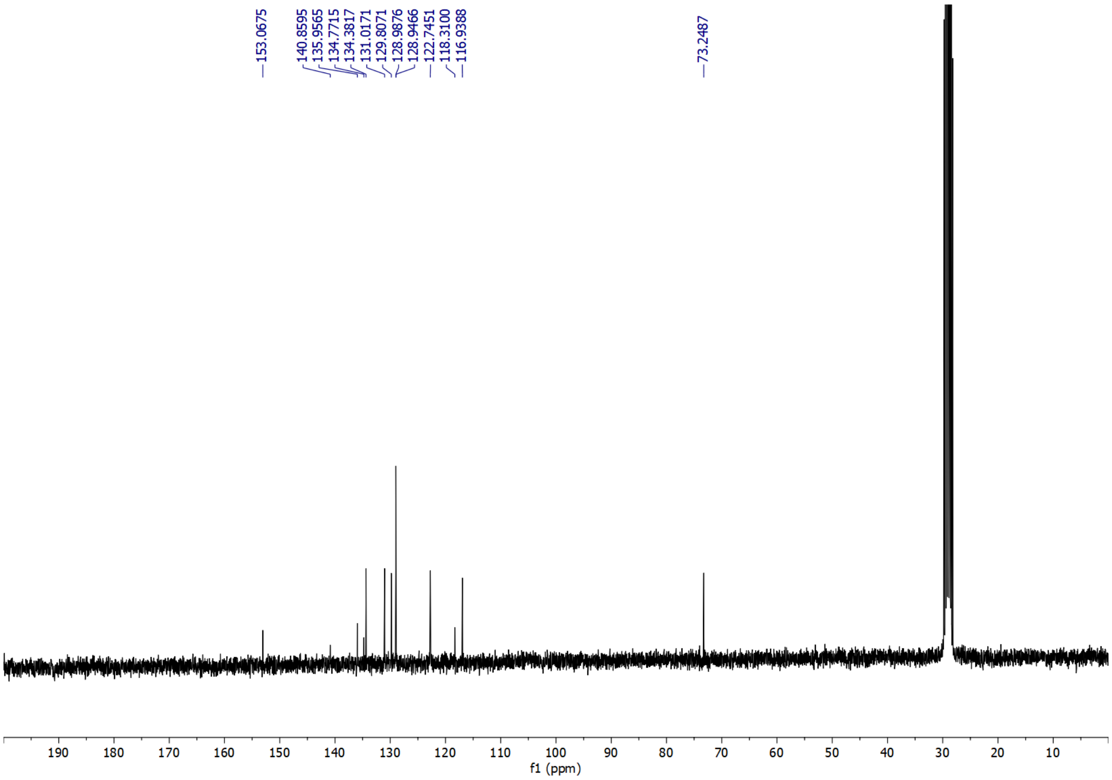

2-(4-Bromophenyl)-3-nitro-2*H*-chromene (5o)

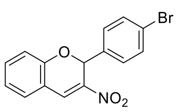

5o

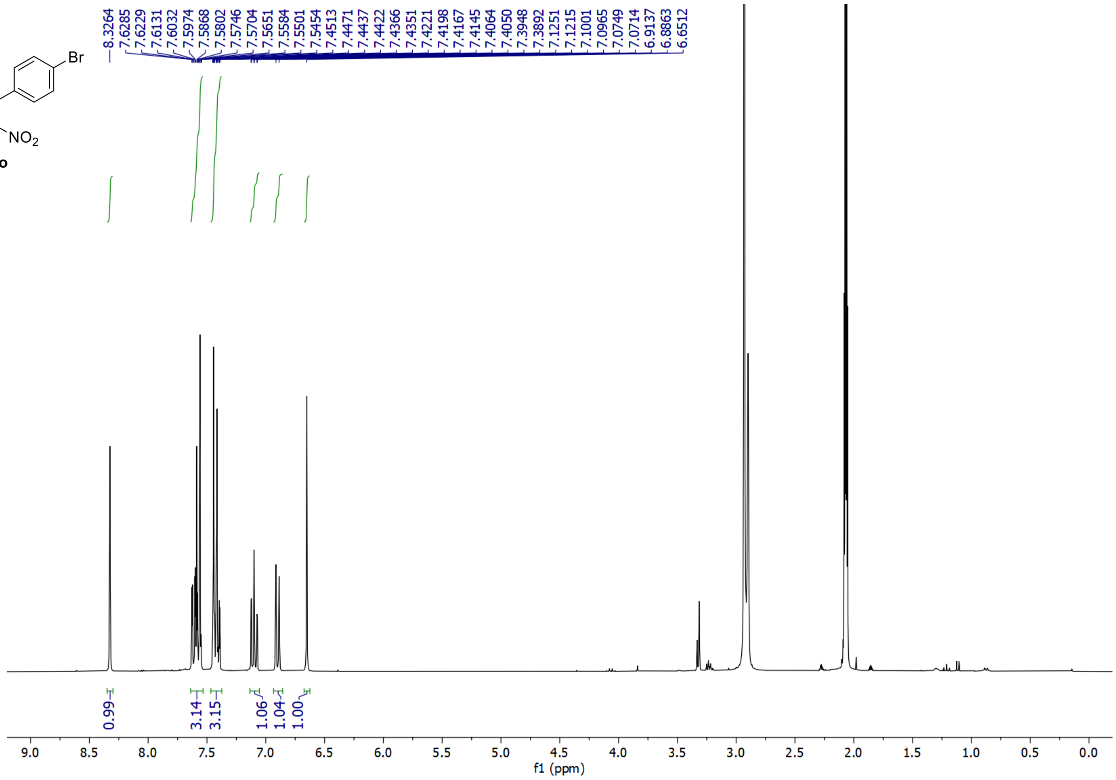

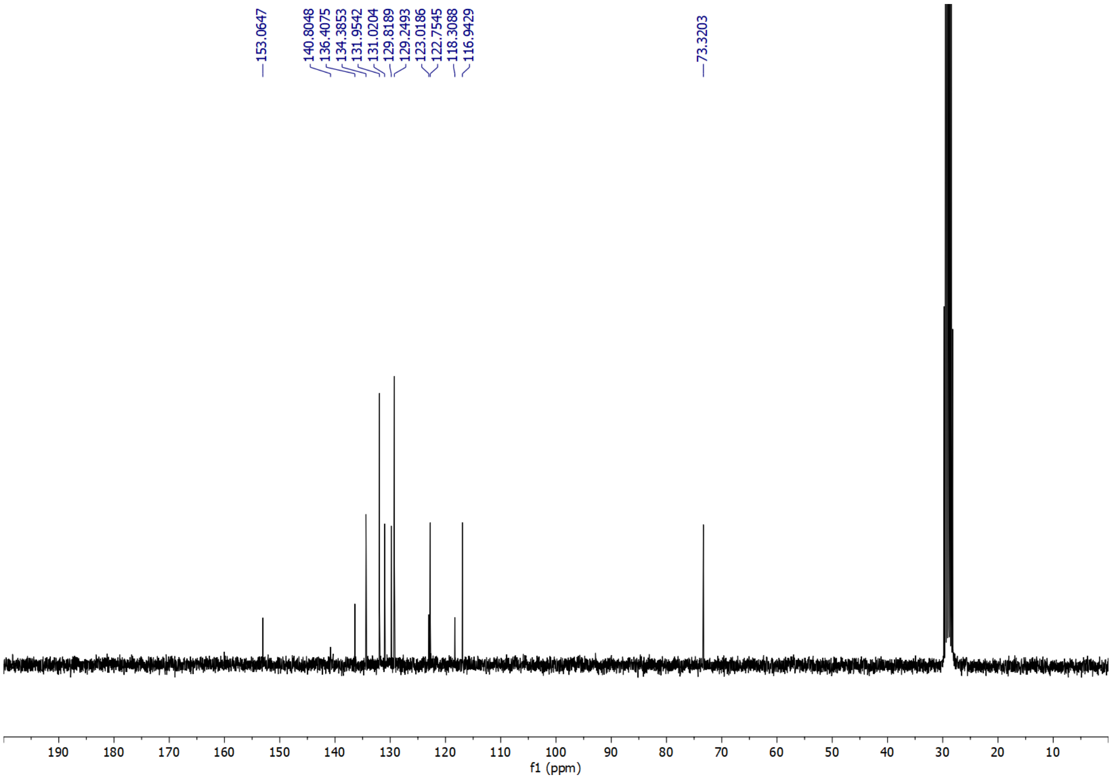

6-Bromo-2-(4-bromophenyl)-3-nitro-2*H*-chromene (5p)

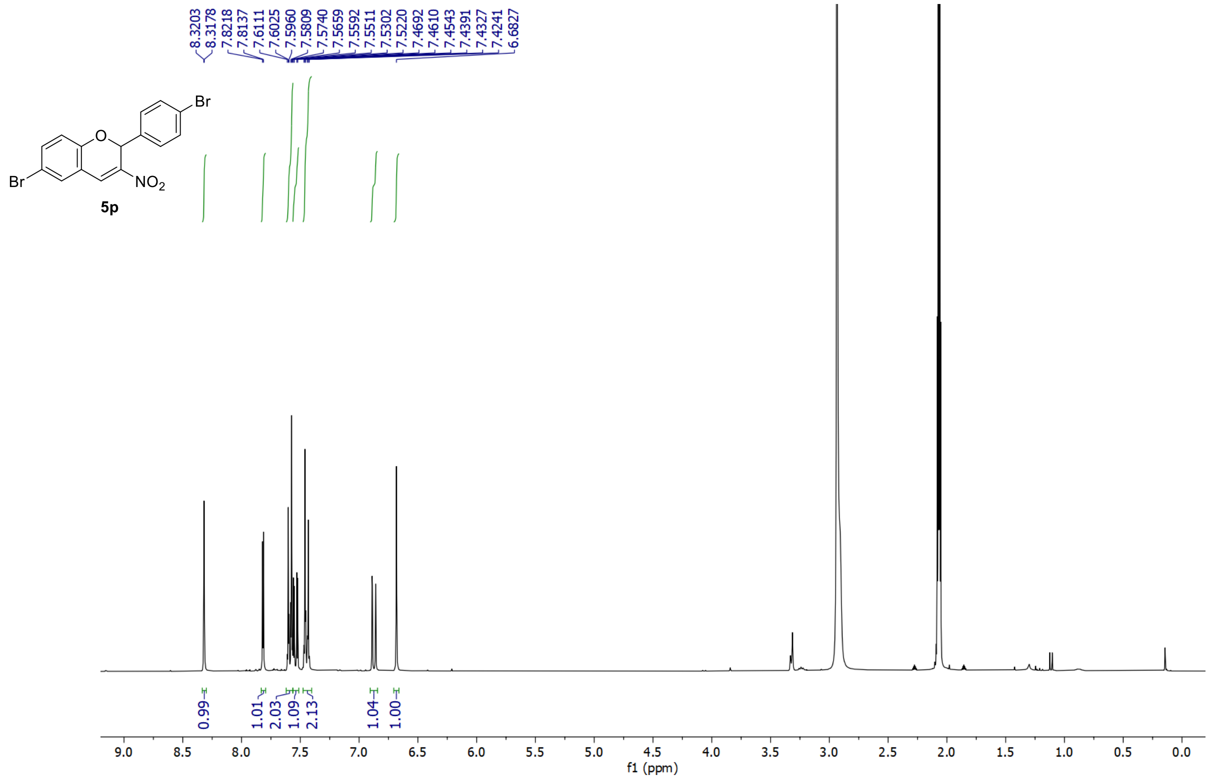

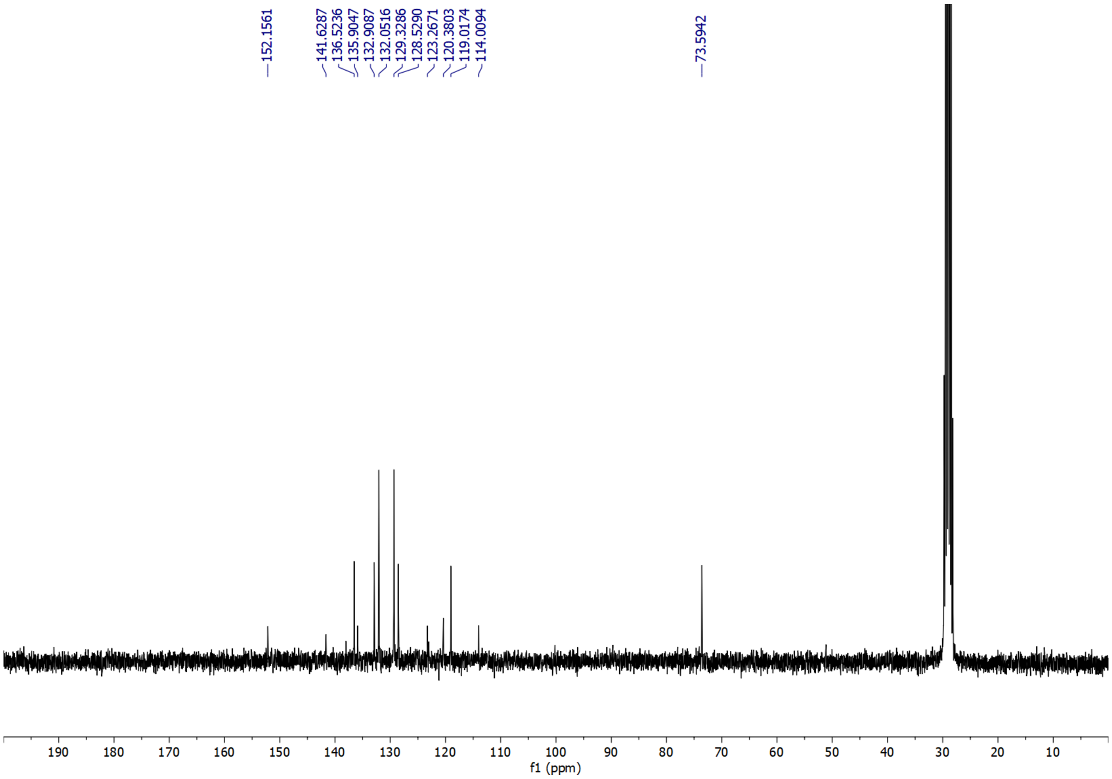

6-Chloro-2-(4-bromophenyl)-3-nitro-2*H*-chromene (5q)

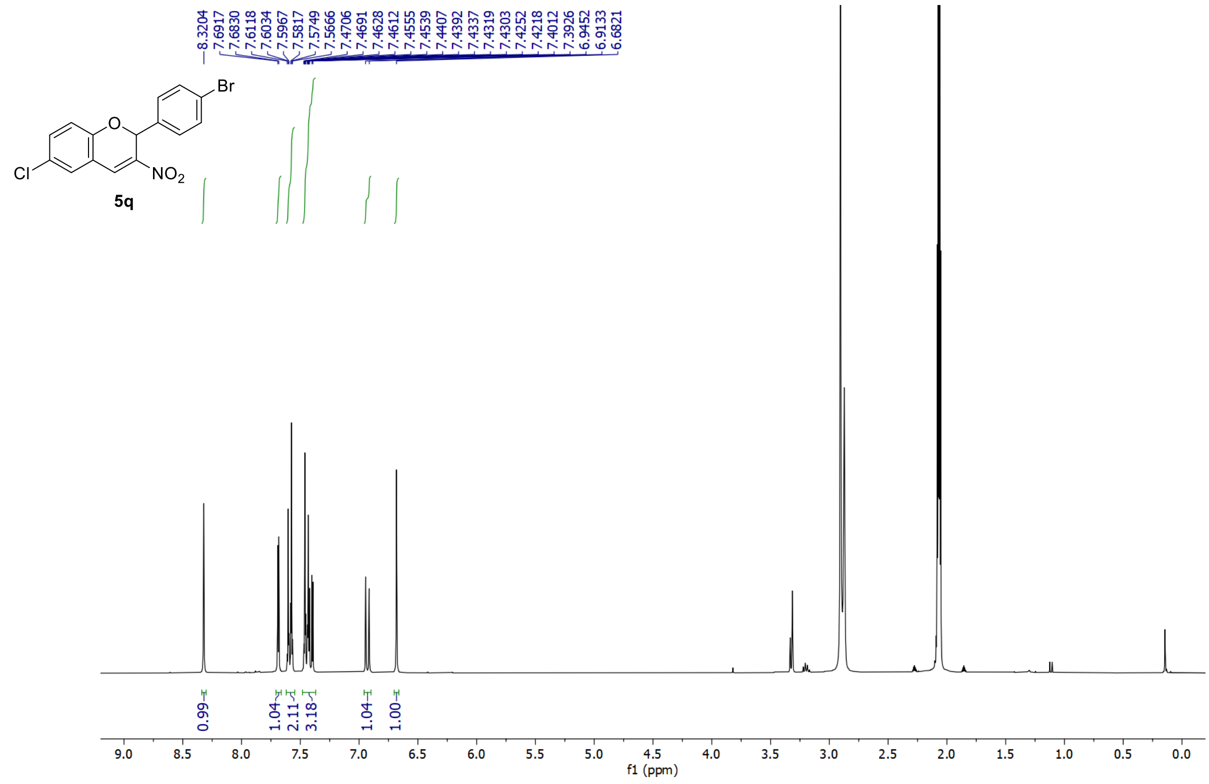

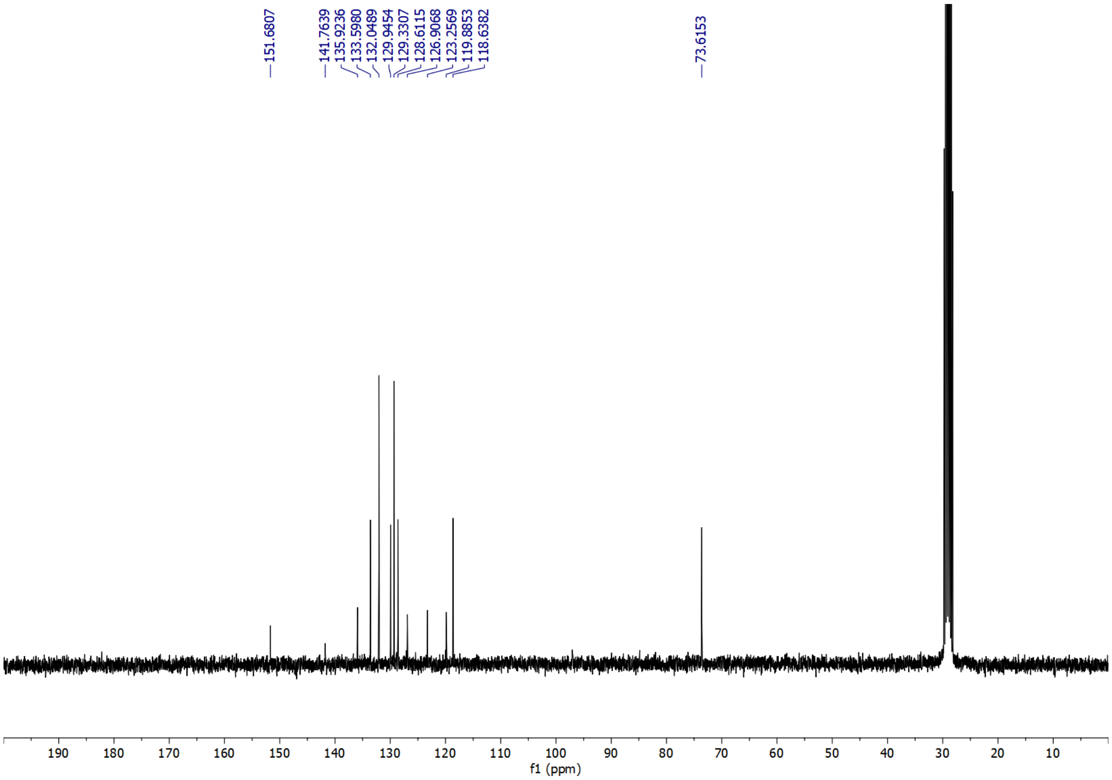

6,8-Dibromo-2-(4-bromophenyl)-3-nitro-2*H*-chromene (5r)

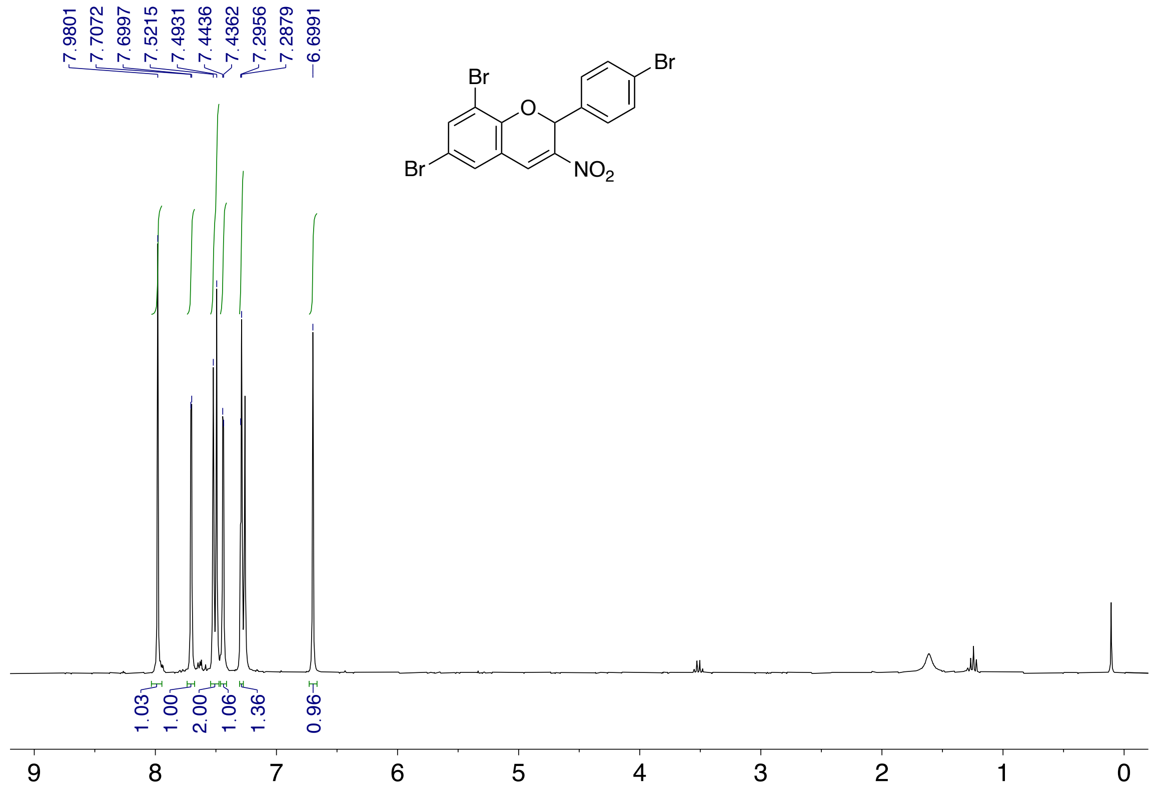

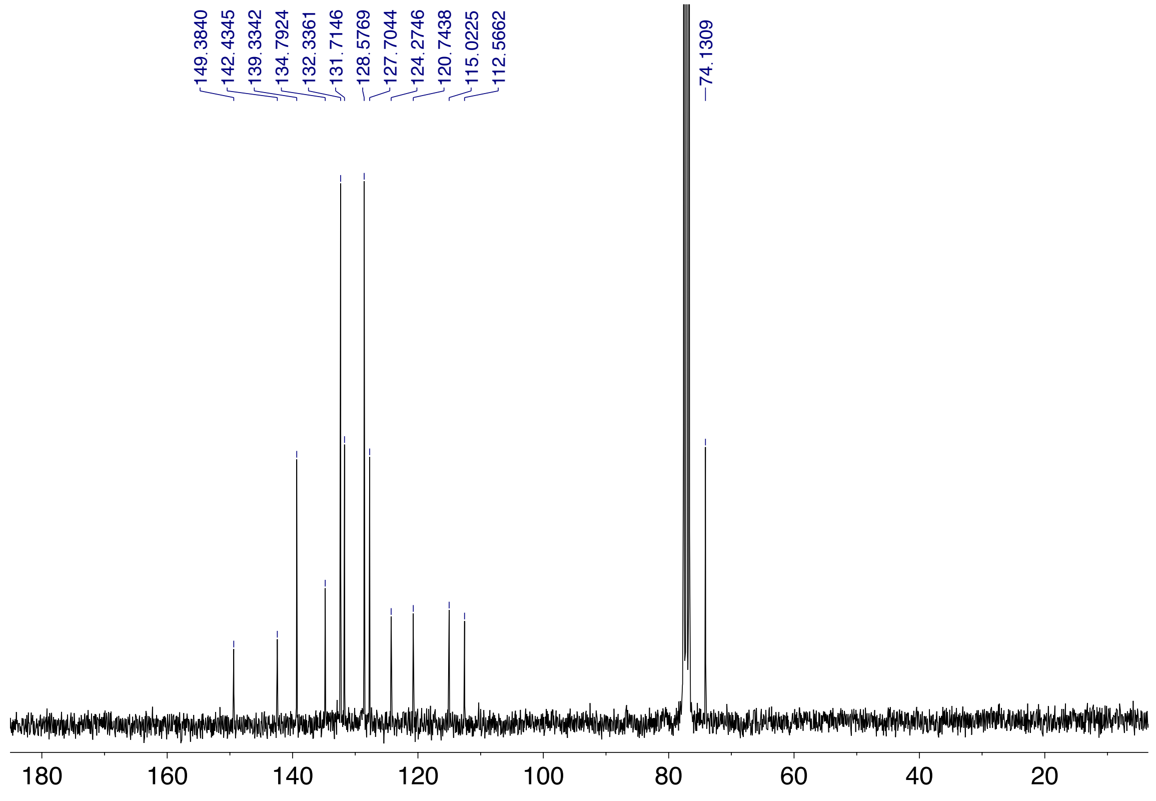

8-Bromo-2-(4-bromophenyl)-6-chloro-3-nitro-2*H*-chromene (5s)

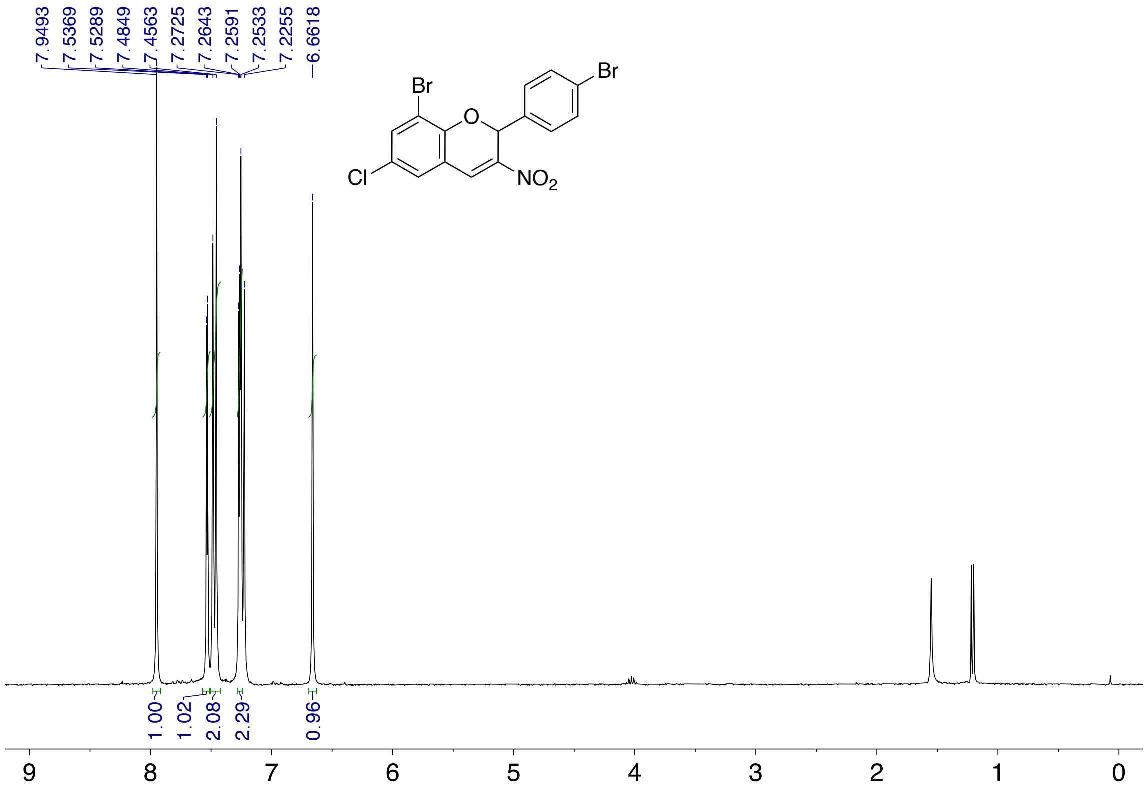

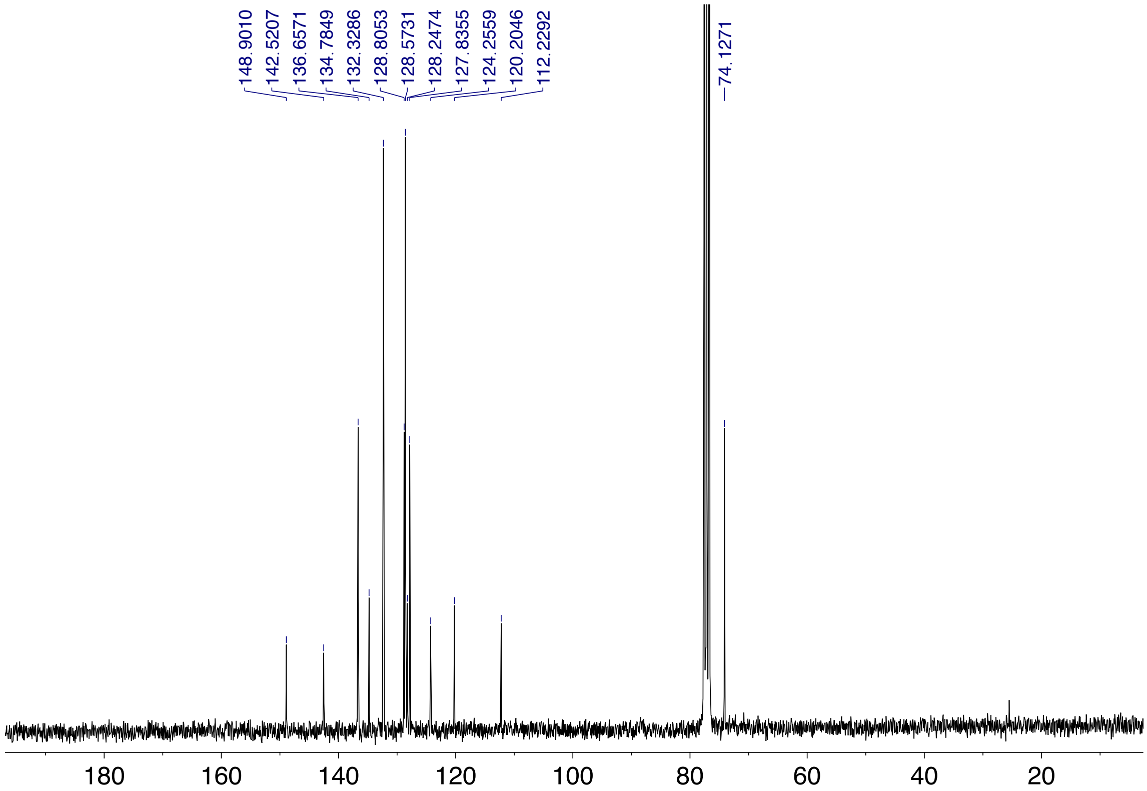

2-(4-Bromophenyl)-6,8-dichloro-3-nitro-2*H*-chromene (5t)

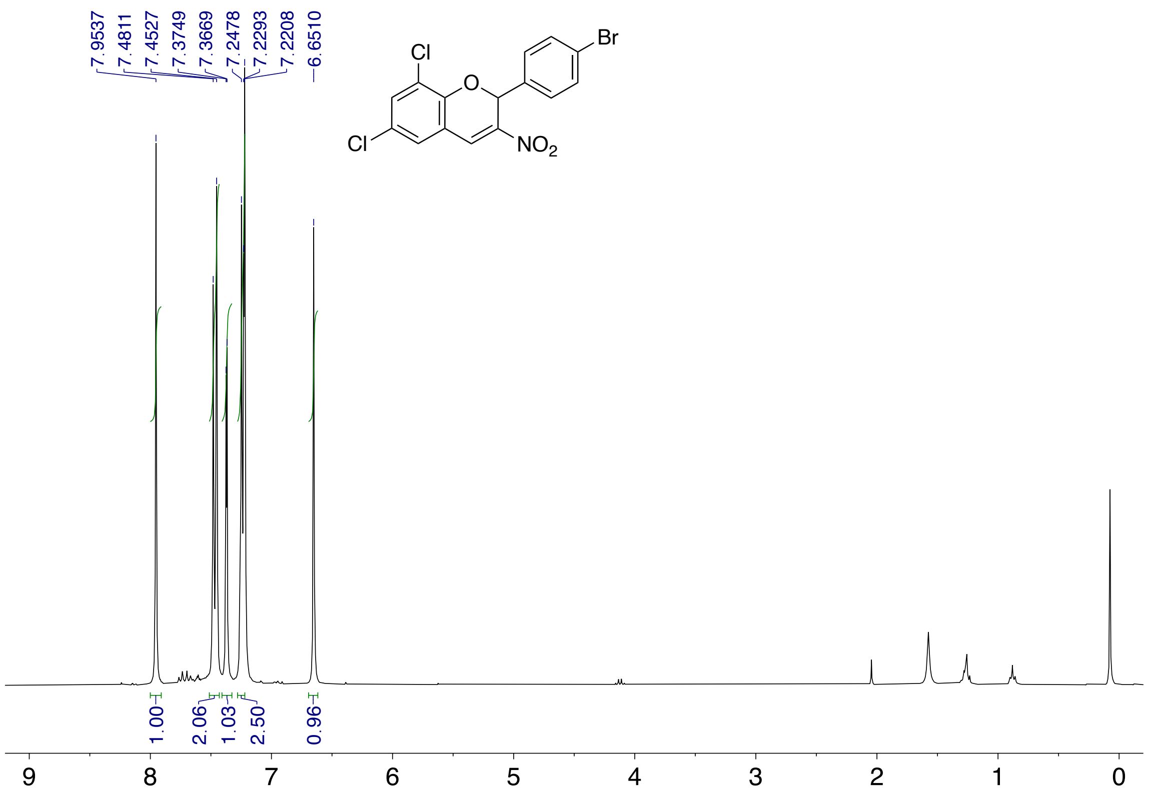
7.9537
7.4811
7.4527
7.3749
7.3669
7.2478
7.2293
7.2208
6.6510
Cl
O
Br
Cl
NO2
1.00
2.06
1.03
2.50
0.96
9
8
7
6
5
4
3
2
1
0

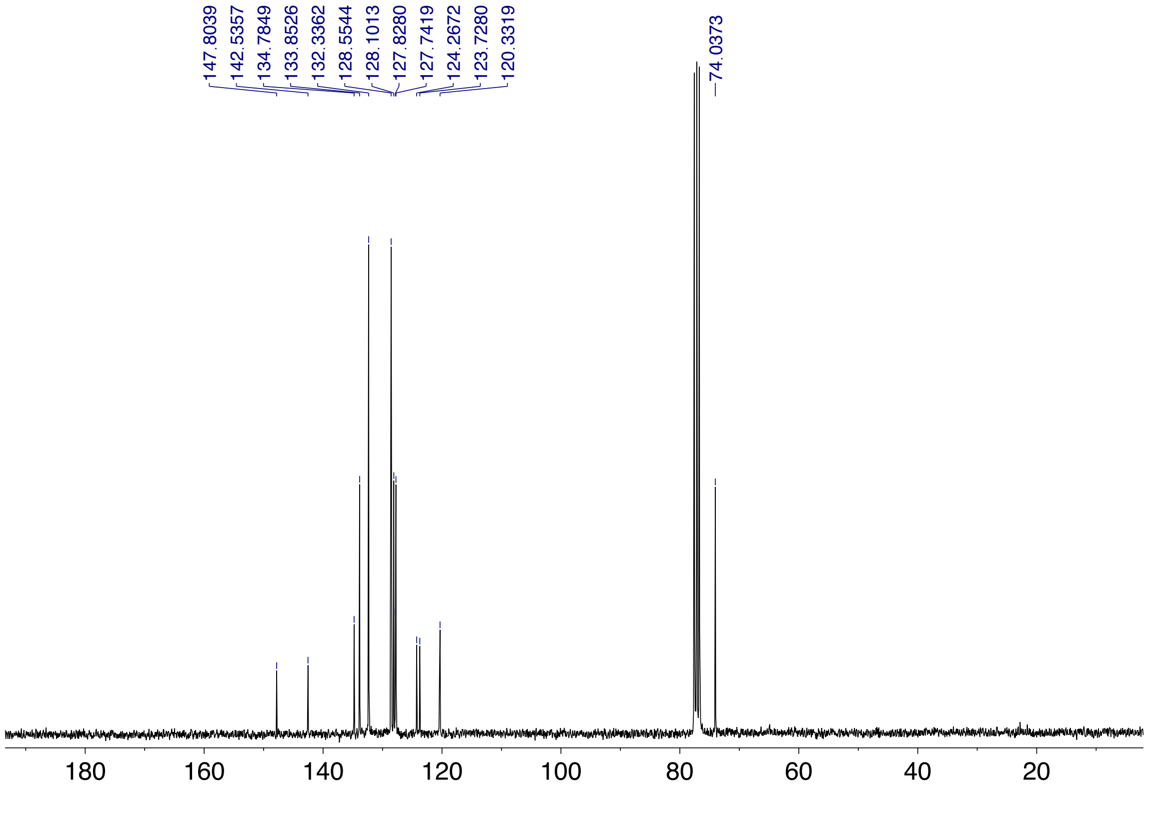
147.8039
142.5357
134.7849
133.8526
132.3362
128.5544
128.1013
127.8280
127.7419
124.2672
123.7280
120.3319
74.0373
180
160
140
120
100
80
60
40
20
